# Discovering Distinct Phenotypical Clusters in Heart Failure Across the Ejection Fraction Spectrum: a Systematic Review

**DOI:** 10.1007/s11897-023-00615-z

**Published:** 2023-07-21

**Authors:** Claartje Meijs, M. Louis Handoko, Gianluigi Savarese, Robin W. M. Vernooij, Ilonca Vaartjes, Amitava Banerjee, Stefan Koudstaal, Jasper J. Brugts, Folkert W. Asselbergs, Alicia Uijl

**Affiliations:** 1 Julius Center for Health Sciences and Primary Care, University Medical Center Utrecht, Utrecht University, Utrecht, the Netherlands; 2 Helmholtz Zentrum München GmbH - German Research Center for Environmental Health, Institute of Computational Biology, Neuherberg, Germany; 3 Department of Cardiology, Amsterdam UMC, Vrije Universiteit Amsterdam, Amsterdam Cardiovascular Sciences, Amsterdam, the Netherlands; 4 Division of Cardiology, Department of Medicine, Karolinska Institutet, Stockholm, Sweden; 5 Department of Nephrology and Hypertension, University Medical Centre Utrecht, Utrecht University, Utrecht, The Netherlands; 6 Health Data Research UK London, Institute for Health Informatics, University College London, London, UK; 7 Department of Cardiology, Green Heart Hospital, Gouda, the Netherlands; 8 Department of Cardiology, Thoraxcenter, Erasmus MC University Medical Center, Rotterdam, The Netherlands; 9 Department of Cardiology, Amsterdam University Medical Centers, University of Amsterdam, Amsterdam, The Netherlands

**Keywords:** Heart failure, Machine learning, Clustering, Phenotyping, Precision medicine

## Abstract

**Review Purpose:**

This systematic review aims to summarise clustering studies in heart failure (HF) and guide future clinical trial design and implementation in routine clinical practice.

**Findings:**

34 studies were identified (*n* = 19 in HF with preserved ejection fraction (HFpEF)). There was significant heterogeneity invariables and techniques used. However, 149/165 described clusters could be assigned to one of nine phenotypes: 1) young, low comorbidity burden; 2) metabolic; 3) cardio-renal; 4) atrial fibrillation (AF); 5) elderly female AF; 6) hypertensive-comorbidity; 7) ischaemic-male; 8) valvular disease; and 9) devices. There was room for improvement on important methodological topics for all clustering studies such as external validation and transparency of the modelling process.

**Summary:**

The large overlap between the phenotypes of the clustering studies shows that clustering is a robust approach for discovering clinically distinct phenotypes. However, future studies should invest in a phenotype model that can be implemented in routine clinical practice and future clinical trial design.

**Graphical Abstract:**

HF = heart failure, EF = ejection fraction, HFpEF = heart failure with preserved ejection fraction, HFrEF = heart failure with reduced ejection fraction, CKD = chronic kidney disease, AF = atrial fibrillation, IHD = ischaemic heart disease, CAD = coronary artery disease, ICD = implantable cardioverter-defibrillator, CRT = cardiac resynchronization therapy, NT-proBNP = N-terminal pro b-type natriuretic peptide, BMI = Body Mass Index, COPD = Chronic obstructive pulmonary disease.

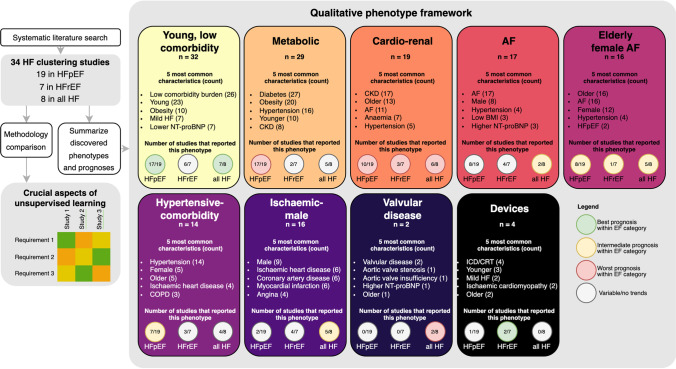

**Supplementary Information:**

The online version contains supplementary material available at 10.1007/s11897-023-00615-z.

## Introduction

Heart failure (HF) is a heterogeneous, chronic syndrome with high morbidity and high mortality, with 10–20% of patients rehospitalised for HF within 1 year and less than 50% of patients surviving 5 years after diagnosis [1, 2]. The prevalence of HF is only expected to increase with an aging general population [3]. Left ventricular ejection fraction (EF) plays a central role in the diagnosis, prognosis, and treatment indication for patients with HF. The European Society of Cardiology (ESC) differentiates EF between HF with reduced EF (HFrEF; EF ≤ 40%), HF with mildly reduced EF (HFmrEF; EF 41–49%), and HF with preserved EF (HFpEF; EF ≥ 50%) [4].

At both ends of the EF spectrum there are limitations in the treatment of patients, which indicates there could be potential for personalisation of care. Treatment of HF follows a “one-size-fits-all” approach, with four main treatments that should be considered for patients with HFrEF. However, with this multitude of evidence-based therapies, an aging population and multimorbidity the management of these patients is complicated [5]. Currently, prioritisation or sequencing of guideline directed medical therapy is lacking, yet personalisation of treatment strategies could be an option for these patients [6].

Only sodium-glucose co-transporter 2 inhibitor (SGLT2i) have demonstrated benefit in patients with HFpEF [7, 8]. Overall, there have been disappointing neutral trial results for patients with HFpEF [9]. The inconclusive trial results in patients with HFpEF might be a consequence of increased underlying heterogeneity in patients with higher LVEF. Yet, there could be subgroups of patients that would benefit from some therapies. This indicates that personalisation is a key concept that could be implemented across the EF spectrum.

Given the high variation in pathophysiology, symptoms, and comorbidities among HF patients, there is significant potential for personalized care. To address the above-mentioned issues, there has been a surge of studies that aimed to describe the heterogeneity of HF patients in a more multidimensional manner, using clustering to characterize phenotypical subgroups.

Unsupervised clustering analysis is a machine learning algorithm that can classify patients according to patient characteristics. Cluster analysis is especially suitable for subgroup discovery when dealing with unknown and complex relationships between variables, as these relationships do not have to be pre-specified to be modelled correctly. A series of clustering studies has been instigated since Shah et al. in 2015 used clustering, which they termed “phenomapping”, to identify clusters of patients with HFpEF [10]. The hypothesis is that increased patient heterogeneity could lead to dilution of beneficial treatment effects.

There is a wide variety of clustering studies in HF, using different clustering methods, identifying variables and HF populations, which makes it difficult to compare these studies. To date, several reviews have discussed clustering, in particular in HFpEF, yet results have not yet been synthesised in a systematic review [11–13]. This systematic review aims to examine and compare the methodology and results of clustering studies that are performed in patients with HF. A comprehensive summary of the clustering studies can shed light on the utility of clustering for patients with HF and the usefulness of corresponding phenotype cluster models, and could help shape future research on treatment personalisation for patients with HF.

## Methods

The review protocol was previously specified and registered in PROSPERO (CRD42022362925). The Preferred Reporting Items for Systematic Reviews and Meta-Analyses (PRISMA) was used to ensure transparent reporting of review methods.

### Eligibility Criteria

Randomised clinical trials and observational studies (cross-sectional, cohorts, registries, and electronic health records) reporting on unsupervised clustering analysis in HF were considered for inclusion. Patients had to be diagnosed with HF, HFrEF, HFmrEF or HFpEF, subpopulations of HF were excluded (e.g. patients with HF and diabetes or patients with HF and destination therapy left ventricular assist devices). Studies were also excluded if the aim of the article was not to define and describe phenotypes within patients with HF or if the analysis did not include unsupervised clustering methods. Clustering studies based on symptoms were excluded. Studies were excluded if they were review articles or case reports. Only studies conducted after 1 January 2010 were considered for synthesis to include contemporary studies on HF and machine learning techniques. The language was restricted to English or Dutch.

### Literature Search

We included relevant search terms for HF, including HFrEF, HFmrEF, HFpEF. In addition, we searched for clustering methods, general terms such as “machine learning” and “clustering analysis” were combined with specific clustering methods such as “latent class analysis”, “hierarchical clustering” and “phenomapping”. Last, we included the outcome of clustering methods such as “clusters”, “phenogroups” and “subgroups”. MeSH terms that were relevant were included. All searches were combined using the Boolean Operators “AND” and “OR”. The search was conducted in two databases: PubMed and EMBASE. The search strategy was conducted on 13 October 2022. A detailed search strategy can be found in Supplementary Table S1.

Final consensus on eligibility, based on title/abstract and full text screening, was reached by two independent reviewers (CM and AU) using the Rayyan web tool.

### Data Extraction and Synthesis

Data was extracted from the included articles according to the following characteristics: 1) general information (year of publication, author, data source), 2) study characteristics (sample size, age and sex distribution), 3) characteristics of clustering (method, number of variables, number of clusters, external validation), 4) data on outcome (identifying variables for each cluster, morbidity and mortality outcomes). A proposed qualitative cluster framework was created summarizing similarities and differences between the cluster models. This framework was developed based on phenotype patterns that we could identify across the different clustering studies. Within the framework characteristic frequency was quantified. Additionally, the characteristics and proportions and prognoses of most common clusters are discussed. Data was extracted by one reviewer (CM) and checked by a second reviewer (AU).

### Quality Assessment

To assess quality of the clustering studies, the methodology of all studies was compared. We consulted the scoping review of Hond et al*.* [14•], and two practical guidelines on clustering to create a comparison structure that contains most crucial aspects of unsupervised learning (Supplementary Table S2) [14•, 15, 16]. The methodology comparison is structured into three phases: 1) preparation, collection, and checking of the data, 2) development of the model, and 3) validation of the model.

## Results

### Literature Search

**Fig. 1:**
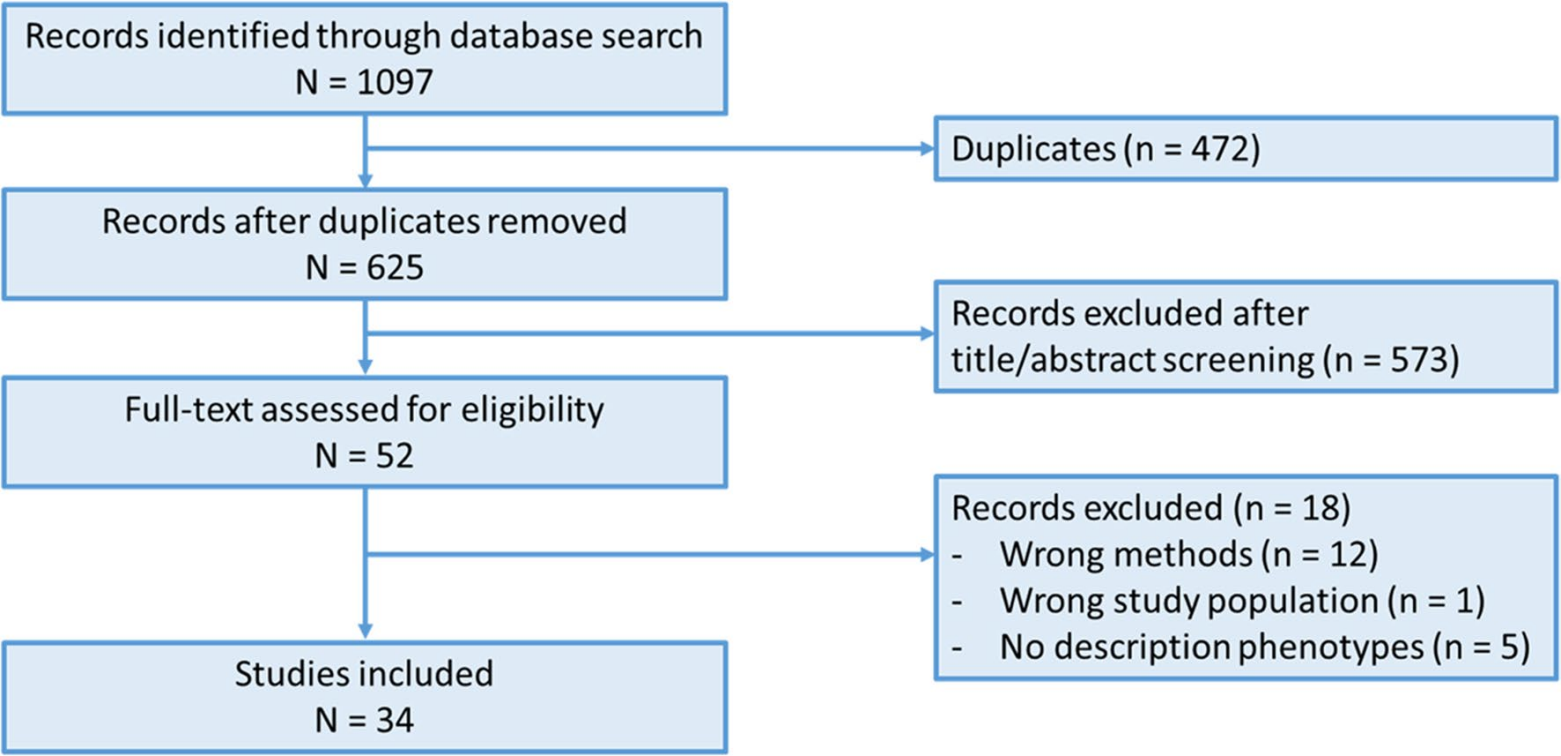
Study flow of the literature search and study selection

A total of 1097 studies were identified in PubMed and EMBASE, of which 472 studies were excluded as duplicates. Studies (*n* = 625) were screened on title/abstract and 52 were selected for full-text review. Of these, 18 were excluded based on wrong methods (i.e. supervised clustering or prediction modelling), wrong study population (i.e. also including non-HF participants) or a missing description of phenotypes (i.e. missing outcome). In total, 34 studies were included in the systematic review (Fig. 1).

### Study Characteristics

We found 34 eligible clustering studies that were performed between 2012 and 2022, and used varying datatypes, clustering methods, and sample sizes (Table 1) [10, 17–33, 34•, 35–38, 39•, 40–42, 43•, 44–49]. Clustering techniques that were used included hierarchical clustering (*n* = 14) [10, 22, 24, 25, 27, 28, 31, 34•, 36, 39•, 40, 45, 47, 49], LCA (*n* = 10) [17, 21, 26, 32, 33, 35, 37, 43•, 46, 48], PAM (*n* = 5) [19, 29, 30, 34•, 38], k-means clustering (*n* = 5) [23, 34•, 41, 42, 44], and model-based clustering (*n* = 3) [18, 20, 34•]. Dataset sizes ranged from 103 patients to 318,384 patients. Datatypes varied between registry-based data (*n* = 6) [19, 26, 27, 42, 43•, 46], cohort data (*n* = 7) [20, 22, 24, 28, 30, 38, 41], EHR data (*n* = 9) [10, 23, 29, 31, 34•, 44, 47–49], and trial data (*n* = 12) [17, 18, 21, 25, 32, 33, 35–37, 39•, 40, 45], using varying variable types for the clustering such as clinical variables (*n* = 31), echocardiographic variables (*n* = 7) [10, 18, 20, 22, 23, 40, 49], biomarkers (*n* = 4) [24, 28, 38, 41], hemodynamic parameters (*n* = 1) [23], and demographic variables (*n* = 1) [27]. The number of variables used for analysis also varied between 8 and 415, and the number of clusters discovered ranged between 2 to 15.

**Table 1:** Summary of key characteristics of clustering studies that were performed in HF patients for phenotype discovery

	Shah [10]	Kao [17]	Segar [18]	Arévalo-Lorido [19]	Hedman [20]	Cohen [21]	Schrub [22]	Harada [23]	Stienen [24]	Gu [25]	Uijl [26]	
HF type	HFpEF	HFpEF	HFpEF	HFpEF	HFpEF	HFpEF	HFpEF	HFpEF	HFpEF	HFpEF	HFpEF	
Publication year	2015	2015	2019	2020	2020	2020	2020	2020	2020	2020	2021	
Dataset	HFpEF Program	I-PRESERVE	TOPCAT	DICUMAP	KaRen	TOPCAT	KaRen	Single center	Media-DHF	Single center	SwedeHF	
Data type	EHR	Trial	Trial	Registry	Cohort	Trial	Cohort	EHR	Cohort	Trial	Registry	
Variable type	Clinical and echocardiographic	Clinical	Clinical and echocardiographic	Clinical	Clinical and echocardiographic	Clinical	Clinical and echocardiographic	Clinical, hemodynamic parameters, and echocardiographic	Biomarkers	Clinical	Clinical	
Number of patients	397	4113	654	103	320	1765	356	350	392	970	6909	
Mean/median age	65	NA	71	79	76	69	76	77	74	70	80	
% Female	62	60	48	60	56	52	57	55	64	42	52	
Clustering method	Hierarchical clustering	LCA	Model-based clustering	PAM	Model based clustering	LCA	Hierarchical clustering	K-means clustering	Hierarchical clustering	Hierarchical clustering	LCA	
Number of variables	67	11	61	23	43	8	55	37	415 (349)	11	10	
Number of clusters	3	6	3	5	6	3	3	4	2	3	5	
Outcome	All-cause mortality, CV-hospitalization and non-CV hospitalization	Event-free survival, all-cause mortality, and CV-hospitalization	All-cause mortality and HF hospitalization	1-year hospitalization and mortality	All-cause mortality and HF-hospitalization	6-year HF hospitalization or all-cause mortality	All-cause mortality and HF hospitalization	Cardiac events	CV death and CV hospitalizations	5-year all-cause mortality and HF hospitalization	All-cause mortality, CV mortality, non-CV mortality, HF hospitalization	
External validation	None	Prognosis and treatment response validation with external validation cohort	Outcome validation in RELAX cohort	None	None	None	None	Outcome validation with internal validation cohort	None	None	CHECK-HF	
	Casebeer [27]	Woolley [28]	Nouraei [29]	Perry [30]	Fayol [31]	Murray [32]	Choy [33]	Banerjee [34•]	Kao [35]	Ahmad [36]	Ferreira [37]	Tromp [38]
HF type	HFpEF	HFpEF	HFpEF	HFpEF	HFpEF	HFpEF	HFpEF	HFpEF	HFrEF	HFrEF	HFrEF	HFrEF
Publication year	2021	2021	2021	2021	2022	2022	2022	2022 (pre-print)	2012	2014	2018	2018
Dataset	MAPD	BIOSTAT-CHF	Single center	Single center	Single center	ASCEND-HF	TOPCAT	THIN	BEST	HF-ACTION	EMPHASIS-HF	BIOSTAT-CHF
Data type	Registry	Cohort	EHR	Cohort	EHR	Trial	Trial	EHR	Trial	Trial	Trial	Cohort
Variable type	Demographic and clinical	Biomarkers	Clinical	Clinical	Clinical	Clinical	Clinical	Clinical	Clinical	Clinical	Clinical	Biomarkers
Number of patients	1515	429	196	889	928	812	1540	188.799	1121	1619	2279	1802
Mean/median age	73	77	77	61	75	74	NA	NA	NA	59	NA	68
% Female	54	45	56	47	43	51	NA	NA	33	28	24	24
Clustering method	Hierarchical clustering	Hierarchical clustering	PAM	PAM	Hierarchical agglomerative clustering	LCA	LCA	K-means, hierarchical, PAM, mixture modelling	LCA	Hierarchical clustering	LCA	PAM
Number of variables	9	363	18	11	15	11	10	87	7	45 (13)	18	92
Number of clusters	3	6	6	7	3	4	3	5	6	4	4	6
Outcome	Treatment and 1-year hospitalization	2-year all-cause mortality and hospitalization	CV mortality	Mortality	All-cause death	30-day hospitalization and mortality	CV mortality, aborted cardiac arrest, or hospitalization for HF	Risk of non-fatal CV diseases, all-cause hospitalization	1-year mortality	Hospitalization and mortality risk	composite of CV death and HF hospitalization	All-cause mortality and HF hospitalisation
External validation	None	None	None	None	None	None	None	CPRD and UK Biobank	Validation of outcomes using the MOCHA population	None	EPHESUS trial	Scottisch independent dataset
	Karwath [39•]	Bouali [40]	de Lange [41]	Ahmad [42]	Tromp [43•]	Nagamine [44]	Gevaert [45]	Gulea[46]	Uszko-Lencer [47]	Zheng [48]	Zhou [49]	
HF type	HFrEF	HFrEF	HFrEF	all HF	all HF	all HF	all HF	all HF	all HF	all HF	all HF	
Publication year	2021	2022	2022	2018	2018	2020	2021	2021	2022	2022	2022	
Dataset	BB-meta-HF	Single-center	Bio-SHiFT	SwedeHF	ASIAN-HF	Single Center	PACT-HF	OLDW	Single-center	Single-center	Single-center	
Data type	Trial	Trial	Cohort	Registry	Registry	EHR	Trial	Registry	EHR	EHR	EHR	
Variable type	Clinical	Clinical and Echocardiographic	Biomarker	Clinical	Clinical	Clinical and symptoms	Clinical	Clinical	Clinical	Clinical	Clinical and echocardiographic	
Number of patients	15659	108	250	44886	6480	25952	1693	318384	603	4063	562	
Mean/median age	64	66	68	76	62	60	77	73	65	69	78	
% Female	24	22	26	38	27	43	49	51	29	66	46	
Clustering method	Hierarchical clustering	Hierarchical clustering	K-means clustering	Random Forest and k-means (partly supervised)	LCA	K-means clustering	Agglomerative hierarchical clustering	LCA	Hierarchical clustering	LCA	Agglomerative hierarchical clustering	
Number of variables	10	17	92	86 (8)	16	NA	14	12	8	12	NA	
Number of clusters	12	2	3	4	5	15	6	5	5	4	5	
Outcome	All-cause mortality	Major adverse cardiac events (MACEs) or death, and RV-remodelling	Event-free survival time	1-year survival	1-year all-cause death or HF hospitalization	Mortality and cardiac events	1-year all-cause death or all-cause rehospitalization	Hospitalization and mortality	Hospital admissions	1-year HF rehospitalization and all-cause mortality	All-cause mortality, CV mortality	
External validation	None	None	none	None	None	None	None	None	None	None	None	

*EHR* Electronic health record; *LCA* Latent class analysis; *PAM* Partitioning around medoids; *CV* Cardiovascular; *HF* Heart failure

### Methodology Comparison

Below, we will discuss a few of the trends that could be observed within the three phases of the quality assessment (Table 2 and Supplementary Table S3).

**Table 2:**
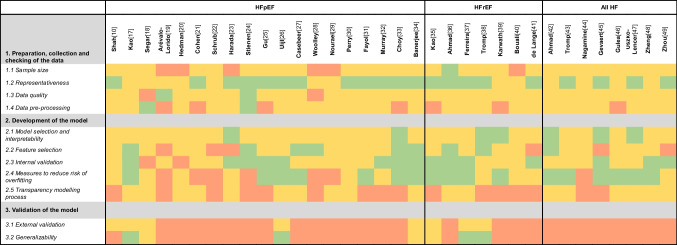
Methodology comparison that summarizes crucial aspects of unsupervised clustering

**Legend.** Structure is based on a scoping review and two practical guides for unsupervised learning [14•, 15, 16]. Green = all requirements for that methodology item have been met, yellow = part of requirements for that methodology item have been met, orange = none of the requirements for that methodology item have been met. Supplementary Table S1 provides a more elaborate description on all methodology requirements, supplementary Table S2 provides a more detailed explanation on the requirement assessment. Unmet requirements can be the result of either not (adequately) performing specific analysis steps or the lack of reporting on details of the analysis steps

#### Preparation, Collection, and Checking of the Data

In over half of the studies the generalizability and representativeness of the participants is evaluated(*n* = 24). However, only rarely sample size requirements are discussed (*n* = 2) [36, 41]. Still, most studies exceeded the threshold of 100 participants for each discovered subgroup (*n* = 29). Description of missingness ranged from not mentioning missing values at all (*n* = 11) [18, 19, 23, 25, 30, 31, 35, 36, 43•, 44, 48] to reporting percentage of missing for each variable (*n* = 12) [10, 20–22, 24, 26, 29, 37, 39•, 40, 46, 47], but also several studies have only given a very global description of the missingness in the dataset usually limited to which variables passed a specific threshold of missingness (*n* = 11) [17, 27, 28, 32, 33, 34•, 38, 41, 42, 45, 49]. From the studies that describe handling of missingness they either performed complete case analysis (*n* = 9) [26, 32, 33, 36–38, 39•, 43•, 46] or imputation (*n* = 13) [10, 17, 18, 20, 22, 24, 29, 34•, 40–42, 48, 49].

#### Development of the Model

Although most studies often described how they selected the number of clusters and helped the reader to interpret the clustering model with either visual aid or with an explanation (*n* = 27), only a small part of the studies provided a description of the advantages and pitfalls of their chosen clustering technique (*n* = 11) [10, 18, 19, 23, 32, 33, 38, 39•, 42, 45, 47]. What is noteworthy is that especially regarding modelling transparency the studies showed low quality, because only rarely the clustering algorithm are being shared (*n* = 9) [17, 23, 26, 34•, 37, 42, 43•, 46, 48], and the code or pipeline was never provided. When it comes to feature selection, part of the studies used all variables available or seem to have used all variables available, as they do not mention feature selection (*n* = 18), some studies select features a priori (i.e., based on clinician perspective, literature or general availability of the variable in the clinic) (*n* = 9) [17, 21, 24, 26, 27, 35, 38, 39•, 47], and other studies use computational approaches to select features (e.g., select features using PCA or correlation coefficient) (*n* = 7) [10, 28, 33, 34•, 36, 42, 44].

#### Validation of the Model

In total, eight studies validated their results in an external validation dataset (Table 2) [17, 18, 23, 26, 34•, 35, 37, 38]. Two of the studies that performed external validation did this with a dataset that was either a subset from the same original dataset or within a dataset that was from the same country, time period, and healthcare setting as the development cohort [10, 34•]. The other studies used external data from different time period, place, or healthcare setting [17, 18, 26, 35, 37, 38]. In the external validation, it was found that phenotypes in the validation cohort had similar outcomes or similar group sizes, depending on whether follow-up data was available.

### Phenotype Comparison

Of the 165 described phenotypes, 149 could be assigned to a proposed qualitative framework of nine phenotypes that transcended studies and EF subtypes (Table 3): 1) young-low comorbidity burden phenotype (*n* = 32); 2) metabolic phenotype (*n* = 29); 3) cardio-renal phenotype (*n* = 19); 4) AF phenotype (*n* = 17); 5) elderly female AF phenotype (*n* = 16); 6) hypertensive-comorbidity phenotype (*n* = 14); 7) ischaemic-male phenotype (*n* = 16); 8) valvular disease phenotype (*n* = 2); and 9) a devices phenotype (*n* = 4). The prevalence of the phenotype characteristics of these nine phenotypes are quantified in Table 4.

**Table 3:**
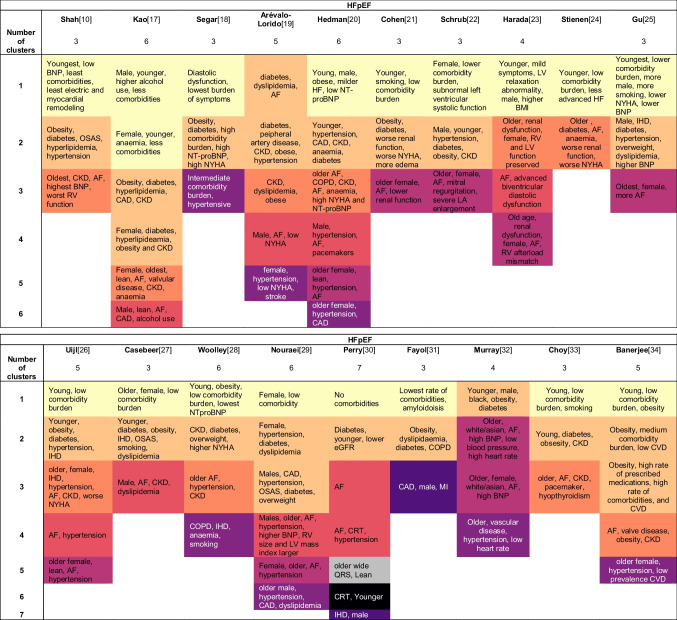

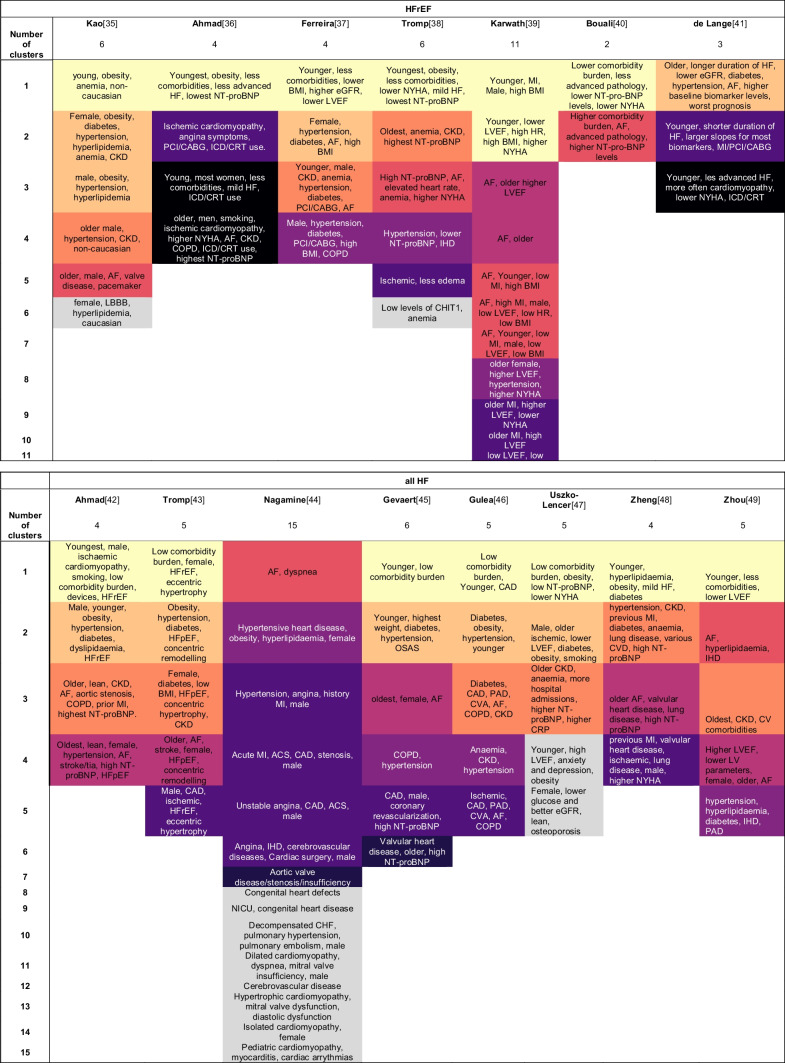
Phenotype key characteristics for each study

Phenotypes that show comparable characteristics are highlighted in the same colour. Yellow: young-low comorbidity phenotype; light orange: diabetic-obesity phenotype; dark orange: cardio-renal phenotype; red: AF phenotype; purple (dark letters): old female phenotype; purple (white letters): hypertensive phenotype; dark violet: ischaemic-male phenotype; dark blue: valvular disease phenotype; black: devices phenotype, grey: other. *AF* Atrial fibrillation; *BMI* Body mass index; *BNP* B-type natriuretic peptide; *CABG* Coronary artery bypass graft; *CAD* Coronary artery disease; *CHF* Congestive heart failure; *CHIT1* Chitotriosidase; *CKD* Chronic kidney disease; *COPD* Chronic obstructive pulmonary disease; *CRT* Cardiac resynchronization therapy; *CVA* Cerebral vascular accident; *CVD* Cardiovascular disease; *eGFR* Estimated glomerular filtration rate; *HF* Heart failure; *ICD* Implantable cardioverter-defibrillator; *IHD* Ischemic heart disease; *LA* Left atrial; *LV* Left ventricle; *LBBB* Left bundle branch block; *LVEF* Left ventricle ejection fraction; *MI* Myocardial infarction; *NICU* Neonatal intensive care unit; *NYHA* New York heart association; *OSAS* Obstructive sleep apnea syndrome; *PAD* Peripheral artery disease; *PCI* Percutaneous coronary intervention; *RV* Right ventricle

**Table 4:**
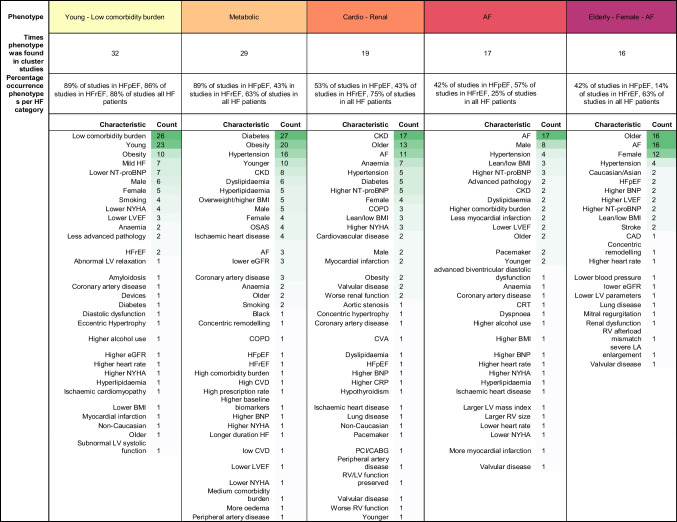

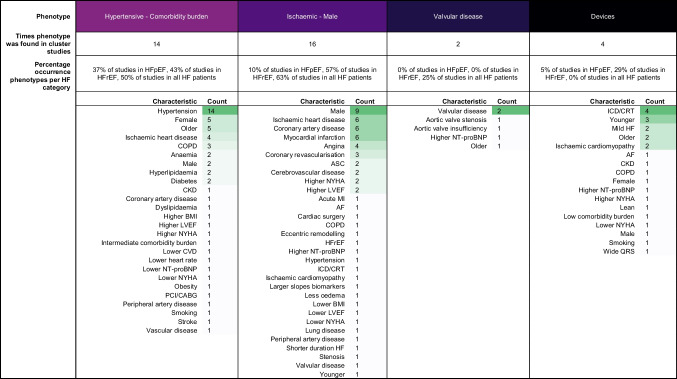
Frequency of phenotype characteristics of the nine most common phenotypes

*AF* Atrial fibrillation; *BMI* Body mass index; *BNP* B-type natriuretic peptide; *CABG* Coronary artery bypass graft; *CAD* Coronary artery disease; *CHF* Congestive heart failure; *CHIT1* Chitotriosidase; *CKD* Chronic kidney disease; *COPD* Chronic obstructive pulmonary disease; *CRT* Cardiac resynchronization therapy; *CVA* Cerebral vascular accident; *CVD* Cardiovascular disease; *eGFR* Estimated glomerular filtration rate; *HF* Heart failure; *ICD* Implantable cardioverter-defibrillator; *IHD* Ischemic heart disease; *LA* Left atrial; *LV* Left ventricle; *LBBB* Left bundle branch block; *LVEF* Left ventricle ejection fraction; *MI* Myocardial infarction; *NICU* Neonatal intensive care unit; *NYHA* New York heart association; *OSAS* Obstructive sleep apnea syndrome; *PAD* Peripheral artery disease; *PCI* Percutaneous coronary intervention; *RV* Right ventricle

#### Young-low Comorbidity Burden Phenotype

The young low comorbidity burden phenotype could be assigned in 17/19 studies in HFpEF, 6/7 studies in HFrEF and 7/8 studies in all HF patients. This cluster is characterised by a lower comorbidity burden and younger age, with in addition obesity (*n* = 10), lower NT-proBNP levels (*n* = 7) and milder HF symptoms (*n* = 7). To some extend lower NYHA (*n* = 4) and smoking (*n* = 4) is reported for this phenotype. Sex is not reported consistently; six studies mention more males while five studies mention more females.

#### Metabolic Phenotype

The metabolic phenotype could be assigned in 17/19 studies in HFpEF, 3/7 studies in HFrEF and 5/8 studies in all HF patients. Patients in this phenotype more often have obesity or are overweight, and have diabetes and hypertension. In addition, younger age (*n* = 10); CKD (*n* = 8) and an imbalance of lipids (*n* = 11) are often reported. Several studies observed some form of ischaemia (IHD *n* = 4; CAD *n* = 3). Sex is not reported consistently; five studies mention more males while four studies mention more females.

#### Cardio-renal Phenotype

The cardio-renal phenotype could be assigned to 10/19 studies in HFpEF, 3/7 studies in HFrEF and 6/8 studies in all HF patients. Patients clustered in this phenotype had CKD or worse renal function, were older and more often had AF. Also more often reported were anemia (*n* = 7), hypertension (*n* = 5) and diabetes (*n* = 5). Several CVDs are observed in this phenotype, including myocardial infarction, valvular disease and coronary artery disease. Sex is not reported consistently; two studies mention more males while four studies mention more females.

#### AF Phenotype

The AF phenotype could be assigned in 8/19 studies in HFpEF, 4/7 studies in HFrEF and 2/8 studies in all HF patients. This phenotype mainly includes patients with AF. Male sex is more reported (*n* = 8) as well as hypertension (*n* = 4). There are inconsistencies between clusters assigned to this phenotype, some studies report younger patients (*n* = 2) whereas others report older patients (*n* = 2).

#### Elderly Female AF Phenotype

The older female phenotype could be assigned in 8/19 studies in HFpEF, 1/7 studies in HFrEF and 5/8 studies in all HF patients. Patients in this phenotype are elderly, have AF and are more often female. In addition, hypertension (*n* = 4), higher BNP/NT-proBNP (*n* = 4) and HFpEF (*n* = 2) are reported.

#### Hypertensive-comorbidity Phenotype

The hypertensive-comorbidity phenotype could be assigned in 7/19 studies in HFpEF, 3/7 studies in HFrEF and 4/8 studies in all HF patients. Patients clustered to this phenotype have hypertension as main comorbidity. In addition, older age (*n* = 5); IHD (*n* = 4) and COPD (*n* = 3) are often reported. Several studies reported anemia, hyperlipidaemia or diabetes (all *n* = 2). Sex is not reported consistently; two studies mention more males while five studies mention more females.

#### Ischaemic-male Phenotype

The ischaemic-male phenotype could be assigned in 2/19 studies in HFpEF, 4/7 studies in HFrEF and 5/8 studies in all HF patients. Patients assigned to this phenotype more often have ischaemic heart disease, CAD or previous myocardial infarction. In addition, angina (*n* = 4); revascularisation (*n* = 3) are more often reported. Several studies also reported higher NYHA (*n* = 2). Nine studies reported more males in this phenotype.

#### Valvular Phenotype

The valvular phenotype could be assigned in 2/8 studies in all HF patients and no studies specifically in patients with HFpEF or HFrEF. Patients assigned to this phenotype more often have valvular disease as main comorbidity. Few other characteristics are reported.

#### Devices Phenotype

The devices phenotype could be assigned in 1/19 studies in HFpEF, 2/7 studies in HFrEF and no studies in all HF patients. Patients assigned to this phenotype more often have implantable devices such as ICD or CRT. In addition, they have milder HF (*n* = 2) and ischaemic cardiomyopathy (*n* = 2). Age is not reported consistently; 3 studies mention younger patients while 2 studies mention older patients.

### Prognosis

The young-low comorbidity phenotype most often had the best prognosis compared to the other subgroups (Table 5). However, this trend was not present in the studies performed on HFrEF patients, where their outcomes were mostly intermediate. The group with the worst outcomes was the cardio-renal phenotype, and this trend can be seen across the EF spectrum. The AF phenotype and male-ischemic phenotype mostly had intermediate prognosis, a trend that was also present in all EF categories. The prognoses of the metabolic phenotype and hypertensive phenotype were highly variant in relation to the other phenotypes, however for the metabolic phenotype it seems that their prognosis in patients with HFpEF is worse than in patients with HFrEF. There was not enough data on the prognosis of the valvular disease phenotype and devices phenotype to discover any trends.

**Table 5:**
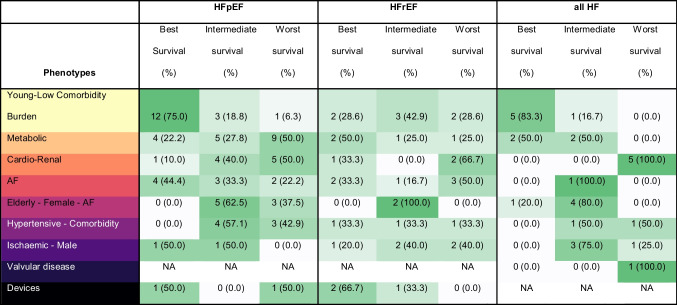
Prognosis of the nine most common phenotypes

*AF* Atrial fibrillation; *HFpEF* Heart failure with preserved ejection fraction; *HFrEF* Heart failure with reduced ejection fraction; *HF* Heart failure

## Discussion

In this systematic review we examined 34 clustering studies in patients with HF, of which 19 studies were exclusively performed in patients with HFpEF. [10, 17–33, 34•, 35–38, 39•, 40–42, 43•, 44–49] Methodologies and phenotypes showed major heterogeneity in the study designs, including the types and sizes of the datasets, clustering algorithms, and selected variables. None of the clustering studies fulfilled all components of the quality assessment, however the degree of methodological limitations differed between the studies. Especially model validation was lacking, only eight studies performed external validation. There was a large overlap in clusters found in the studies, and we identified nine commonly described phenotypes: young-low comorbidity burden; metabolic; cardio-renal; AF; elderly female AF; hypertensive-comorbidity; ischaemic-male; valvular disease; and devices.

### Qualitative Phenotype Framework

Based on the clustering studies we created a qualitative phenotype framework consisting of 9 phenotypes. Two phenotypes were most consistently seen in all clustering studies: the young-low comorbidity burden phenotype and metabolic phenotype. To explain the young-low comorbidity burden phenotype, we hypothesize that part of these patients might have BNP deficiency syndrome as proposed by Shah et al. in 2015 [10]. At least 10 cluster studies reported obesity in this phenotype and it is known that obesity can influence the BNP clearance through higher neprilysin levels and increased renal filtration [50]. Furthermore, especially in the studies in HFpEF, it could indicate that patients in this phenotype have recovered HF after treatment with guideline recommended therapy. Another potential reason for this phenotype could be that these patients simply have less severe/advanced HF, which is in line with the better prognosis trends seen in this phenotype.

For the metabolic phenotype, both obesity and diabetes are prone to occur in HF patients. Obesity has been shown to be associated with adverse hemodynamic changes that predispose to cardiac remodelling and ventricular dysfunction and thus HF, also in the absence of other comorbidities [51]. In addition, diabetes has also been shown to be independently associated with an increased risk of HF, cardiovascular mortality, and HF hospitalization [52]. This phenotype appears to cluster more around patients with HFpEF compared to those with HFrEF, yet can still be found across the EF spectrum. This confirms the notion that HFpEF pathophysiology is more driven by metabolic disturbances and an inflammatory burden [53].

There were three phenotypes that all had AF as one of the main three components, these were the cardio-renal, AF and elderly-female phenotypes. These were included in the qualitative framework as three phenotypes as there were distinct differences between clusters with regards to presence across the EF spectrum and prognosis. Several studies have shown the close relation between AF and HF [54]. What is unique about the AF phenotype is that these patients often are of intermediate age and can be both male or female, in contrast to the elderly-female-AF phenotype. This phenotype also appeared across the EF spectrum, yet there could be differences in the pathophysiology of this phenotype. For example, the prognosis of this phenotype appears to be worse in patients with HFrEF, whereas it appears better in patients with HFpEF. It is proposed that in HFrEF, AF may be a consequence of the HF, whereas in HFpEF, both ventricular and atrial myopathy may develop in parallel [54, 55]. And indeed, several studies reported changes in left ventricle and left atrium parameters. Which can be seen in the elderly-AF-female phenotype, which was more prevalent in patients with HFpEF. The proportion of patients with HF and concomitant AF increases with age, which is very likely observed in this phenotype [56].

In the cardio-renal phenotype we observe the bidirectional interaction between kidney function and HF [57]. Previous studies have shown that CKD is more common in patients with HFpEF, yet might play a larger role in the prognosis of patients with HFrEF [58, 59]. In this review we consistently show a worse survival regardless of EF. In addition, several studies reported anaemia, which could be a consequence of the presence of CKD [60]. Anaemia also independently contributes to worse prognosis in HF [61]. Studies have shown that treating anaemia in HF patients is associated with improvements of NYHA class, symptoms and HF hospitalisations [62, 63].

Two phenotypes occurred more frequently in the studies investigating HFrEF patients: The ischaemic-male and devices phenotypes. Ischaemia is one of the underlying cause of HF, and more often in men, where it is the main cause of HF [64, 65]. Patients in this cluster could have a variety of previous ischaemic diseases in their underlying disease pathology for HFrEF [66].

ICDs are implanted in patients with HF that are at risk for sudden cardiac death or all-cause mortality according to the recommendations in the guidelines as both primary and secondary prevention [4]. Primary prevention is targeted to those patients that have symptomatic HF (NYHA class II-III) of an ischaemic aetiology and LVEF ≤ 35%. Across the studies, this phenotype occurred mainly in HFrEF patients and was also seen in one study with recovered HF patients. Personalisation in ICD placement is a current unmet need [67]. Clustering could potentially play a role in this personalisation.

The hypertensive-comorbidity phenotype was characterised by the absence of obesity and diabetes and presence of comorbidities such as COPD and IHD. There are several diagnostic challenges in COPD and HF as clinical symptoms can be overlapping [68, 69]. Different characteristics could potentially be used to better define this phenotype, such as biomarkers or echocardiographic parameters.

The valvular disease phenotype was a specific phenotype related to hospitalised inpatients described in two studies in EHR data (in- and outpatients) and one based on a clinical trial (tertiary care or quaternary care) [44, 45]. Valve disease is a known aetiology for HF with a very poor prognosis, with the three main diseases aortic stenosis, aortic regurgitation and mitral insufficiency[4].

### Are we There Yet? Precision Medicine for HF

There was significant overlap in the clustering outcomes between the various studies [10, 17–33, 34•, 35–38, 39•, 40–42, 43•, 44–49]. Yet, differences between the clustering studies still exist.. This indicates that there is a lack of precision at least to some extent in the subgroups based on clustering.

We hypothesize that the differences in phenotype descriptions could be due to differences between the data sources, as phenotypes characteristics are relative to their patient population. This limits the reproducibility and generalisability of the cluster models to other patient populations and use in routine clinical care. A potential solution could be readjusting or fine-tuning the current models using site specific information to increase the generalisability.

In addition, it is important to underline that we grouped the clusters based on reported characteristics to one of the nine phenotypes. It could be that there are unreported characteristics that would categorize a cluster to a different phenotype if they were known.

### Implementation and Future Perspective

Ideally, this systematic review could identify one or multiple clustering studies of sufficient quality for implementation in clinical trials or in clinical practice. Due to the high heterogeneity and absence of a gold standard, this is not possible. Nonetheless, the findings of this review suggest that clustering is a suitable and fruitful approach for capturing the underlying heterogeneity of patients with HF.

Future studies should be aware of the methodological caveats of clustering research and take this into account when performing these studies. Efforts should be directed towards improving the development and validation of clustering as machine learning model [70]. Validating existing models could potentially lead to a more precise, valid and reliable phenotyping model that could be implemented in clinical trial design or as a decision tool in daily clinical practice.

Most importantly, we found that clusters transcend across the EF spectrum, indicating that clusters might not be limited to heterogeneity in HFpEF, but could also play a role in HFrEF. There were significant differences based on prognosis that are worth to be explored further. In addition, it has not yet been investigated whether patients could change between clusters over time. Longitudinal data is necessary to uncover any transitions over time.

Current studies should therefore be considered as hypothesis generating. In the future it would be potentially be possible to investigate differences in prognosis and treatment benefit in clinical trials. Differences in prognosis could be used to guide future trial inclusion to optimise and enrich clinical trials. Moreover, patients in trials could be stratified based on clustering models to see whether there are different treatment effects. Currently, there is limited data on treatment heterogeneity across clusters and should be studied further. Investigating this would mean a step forward towards finding beneficial treatment options or strategies on subgroup patient level and in the future on individual patient level.

### Strengths and Limitations

One of the strengths of this systematic review is that both results and methodology of the clustering studies were compared. This gives context to the results and can provide nuance in the discussion on the reliability and validity of the clustering studies. In addition, the large amount of clustering studies and the heterogeneity of their study designs increase the meaningfulness of their similarities regarding their phenotype models. This enables us to quantify the degree of certainty to some extent regarding phenotype characteristics. Moreover, this systematic review showed a general overview of the requirements of unsupervised clustering. In cardiology, clustering is an increasingly common technique for subgroup discovery, and a basic understanding of the strengths, limitations, and pitfalls of clustering can help facilitate critical evaluation of these studies. Furthermore, at this moment we are the first systematic review that has performed rigorous review methodology and that gives an elaborate overview of both the discovered phenotypes and methodology.

In this systematic review, we developed a methodological quality assessment, as current tools suitable for clustering meta-analysis are non-existent and validated guidelines on reviewing clustering studies are lacking. Therefore, a more descriptive approach has been used. To compare methodologies, the quality assessment was based on a scoping review of Hond et al*.* [14•], and two practical guidelines on clustering [14•, 15, 16]. It is important to note that we could not always distinguish between worse performance of a study or only lacking to report certain aspects.

The issue of generalizability across different ethnic or social economic backgrounds in patients with HF has been debated. Some have argued that these differences may lead to variations in the presentation and treatment of HF that require separate subgroup analyses [71]. Currently, there is not enough evidence to support biological differences between different populations, therefore, it may be deemed appropriate to generalize our findings to other populations. Indeed, three studies in an Asian population presented comparable phenotypes as to those with other ethnicities [43•, 48, 49].

Lastly, we grouped the clusters according to the reported characteristics for each study. It could be that there are other underlying characteristics that would change the phenotype assignment.

## Conclusions

There were many differences between the clustering studies regarding the sizes and types of the datasets, variable selection, and algorithms, but they yielded comparable phenotypes which implies that clustering is a fruitful approach for phenotype discovery. Specifically, of the 165 phenotypes that were described, 149 could be assigned to one of nine most common phenotypes: young-low comorbidity burden; metabolic; cardio-renal; AF; elderly female AF; hypertensive-comorbidity; ischaemic-male; valvular disease; and a devices phenotype. These phenotypes are not limited to a particular EF, but rather transcended across the EF spectrum. Comparing methodologies of the studies showed that there was still room for improvement on topics concerning validity and reliability, especially regarding external validation. These methodological aspects limit the current implementation into clinical practice and effort should be directed towards improving the clinical utility of cluster analysis. Altogether, this systematic review is hypothesis generating and lays the groundwork for future research into a more precise and reliable phenotype model that can serve as a stratification and decision tool in clinical trial design and personalised medicine for patients with HF.

### Supplementary Information

Below is the link to the electronic supplementary material.

Supplementary file1 (DOCX 95 kb)
