# Supplementary material for: Discovering Distinct Phenotypical Clusters in Heart Failure Across the Ejection Fraction Spectrum: a Systematic Review
Source: Curr Heart Fail Rep. 2023 Jul 21;20(5):333–49. doi: 10.1007/s11897-023-00615-z (PMC10589200; doi:10.1007/s11897-023-00615-z)
Supplement: Supplementary file 1 — Supplementary file1 (DOCX 95 kb) [file 11897_2023_615_MOESM1_ESM.docx]

**Supplementary Material**

**Table S1. Search strategy**

|  | **Pubmed** | **Studies** |
| --- | --- | --- |
|  | **Date: 12 October 2022** |  |
| #1 | (“heart failure”[MeSH Terms] OR “heart failure”[Title/Abstract] OR “HFpEF”[Title/Abstract] OR “HFmrEF”[Title/Abstract] OR “HFrEF”[Title/Abstract] OR “preserved ejection fraction”[Title/Abstract] OR “mildly reduced ejection fraction”[Title/Abstract] OR “reduced ejection fraction”[Title/Abstract] OR “mid-range ejection fraction”[Title/Abstract] OR “recovered left ventricular ejection fraction”[Title/Abstract] OR “reduced left ventricular ejection fraction”[Title/Abstract] OR “mildly reduced left ventricular ejection fraction”[Title/Abstract] OR “mid-range left ventricular ejection fraction”[Title/Abstract]) AND (2010/01/01:2022/10/12 [Date - Publication]) | 139,715 |
| #2 | (“machine learning”[MeSH Terms] OR “cluster analysis”[MeSH Terms] OR “phenomap*”[Title/Abstract] OR “latent class”[Title/Abstract] OR “unsupervised”[Title/Abstract] OR “hierarchical”[Title/Abstract] OR “unsupervised clustering”[Title/Abstract] OR “model-based clustering”[Title/Abstract] OR “partitioning around medoids”[Title/Abstract] OR “PAM”[Title/Abstract] OR “phenotypic clustering”[Title/Abstract] OR “unsupervised cluster analysis”[Title/Abstract] OR “unsupervised machine learning”[Title/Abstract] OR “latent-class analysis”[Title/Abstract] OR “cluster analysis”[Title/Abstract] OR “clustering analysis”[Title/Abstract] OR “clustering”[Title/Abstract]) AND (2010/01/01:2022/10/12 [Date - Publication]) | 235,060 |
| #3 | (“subgroup*”[Title/Abstract] OR “cluster*”[Title/Abstract] OR “phenotyp*”[Title/Abstract] OR “phenogroup*”[Title/Abstract] OR “distinct groups”[Title/Abstract]) AND (2010/01/01:2022/10/12 [Date - Publication]) | 877,987 |
| #5 | #1 AND #2 AND #3 | 556 |
|  | **Embase** |  |
|  | **Date: 12 October 2022** |  |
| #1 | (“heart failure”:ti,ab,kw OR “HFpEF”:ti,ab,kw OR “HFmrEF”:ti,ab,kw OR “HFrEF”:ti,ab,kw OR “preserved ejection fraction”:ti,ab,kw OR “mildly reduced ejection fraction”:ti,ab,kw OR “reduced ejection fraction”:ti,ab,kw OR “mid-range ejection fraction”:ti,ab,kw OR “recovered left ventricular ejection fraction”:ti,ab,kw OR “reduced left ventricular ejection fraction”:ti,ab,kw OR “mildly reduced left ventricular ejection fraction”:ti,ab,kw OR “mid-range left ventricular ejection fraction”:ti,ab,kw) AND [2010-2022]/py | 250,745 |
| #2 | (“machine learning”:ti,ab,kw OR “cluster analysis”:ti,ab,kw OR “phenomap*”:ti,ab,kw OR “latent class”:ti,ab,kw OR “unsupervised”:ti,ab,kw OR “hierarchical”:ti,ab,kw OR “unsupervised clustering”:ti,ab,kw OR “model-based clustering”:ti,ab,kw OR “partitioning around medoids”:ti,ab,kw OR “PAM”:ti,ab,kw OR “phenotypic clustering”:ti,ab,kw OR “unsupervised cluster analysis”:ti,ab,kw OR “unsupervised machine learning”:ti,ab,kw OR “latent-class analysis”:ti,ab,kw OR “clustering analysis”:ti,ab,kw OR “clustering”:ti,ab,kw) AND [2010-2022]/py | 274,459 |
| #3 | (“subgroup*”:ti,ab,kw OR “cluster*”:ti,ab,kw OR “phenotyp*”:ti,ab,kw OR “phenogroup*”:ti,ab,kw OR “distinct groups”:ti,ab,kw) AND [2010-2022]/py | 1,269,031 |
| #4 | #1 AND #2 AND #3 NOT “conference abstract” | 1,073 |
| #5 | #4 NOT “conference abstract” | 541 |

**Table S2. Description of the crucial aspects of unsupervised learning**[14–16]

| **1. Preparation, collection and checking of the data** | |
| --- | --- |
| *1.1 Sample size* | 1) Reporting of sample size requirements and 2) at least 100 events or participants per outcome (phenotype) |
| *1.2 Representativeness* | Representation of real-world heterogeneity and diversity, requiring 1) reporting of data collection and baseline characteristics, and 2) reporting where representation is missing |
| *1.3 Data quality* | 1) Description of missing data, 2) consideration of potential errors in measurements, and their underlying mechanisms, 3) reporting prospective or retrospective collection and 4) prospective collection is preferred |
| *1.4 Data pre-processing* | Detailed description of 1) removing outliers, re-coding or transforming, standardization, and 2) imputation preferred over complete case analysis, and 3) detailed description of imputation of missing data (MAR, MCAR, MNAR) |
| **2. Development of the model** | |
| *2.1 Model selection and interpretability* | 1) Motivation for model selection clearly articulated including potential risks, 2) facilitating interpretability of model, 3) careful selection of number of phenotypes |
| *2.2 Variable selection* | 1) Description of variable selection and variable meaning, 2) removal of highly correlated variables, and 3) limiting number of variables |
| *2.3 Internal validation* | 1) Report measures of internal validation such as reliability or stability of phenotypes, 2) meaningfulness of found phenotypes, and 3) comparable phenotype sizes with >5% of total number of participants in each phenotype |
| *2.4 Measures to reduce risk of overfitting* | 1) Ratio sample size and candidate variables at least 30:1, and 2) penalizing model complexity |
| *2.5 Transparency modelling process* | 1) Reporting software packages and versioning, 2) publishing of the code for the complete model building pipeline, and 3) providing phenotype model algorithm for application |
| **3. Validation of the model** | |
| *3.1 External validation* | 1) Validation of model in different dataset, and 2) validation performed by independent researchers from other institutions or settings |
| *3.2 Generalizability* | 1) Validate generalizability of the model using external data from different time period, place, or healthcare setting, 2) validate in dataset with sufficient sample size |

**Table S3: detailed description of the methodology assessment using the framework described in Table S2**. According to the number of methodological aspects the studies have reported on or have performed correctly, **Table 2** was constructed to visually summarize the methodology assessment.

| **1. Preparation, collection and checking of the data** | | **Shah**[10] | **Kao**[17] | **Segar**[18] | **Arévalo-Lorido**[19] | **Hedman**[20] | **Cohen**[21] | **Schrub**[22] |
| --- | --- | --- | --- | --- | --- | --- | --- | --- |
| *1.1 Sample size* | 1) Reporting of sample size requirements and 2) at least 100 events or participants per outcome (subgroup) | 1) no, failed reporting of sample size requirements, 2) yes, they have 3 subgroups and they have >300 participants | 1) no report of sample size requirements, and 2) yes, enough participants for all subgroups as they have >600 participants | 1) no, sample size requirements not discussed, and 2) yes, >300 participants | 1) no, report of sample size requirements is missing. 2) no, less than 500 participants | 1) no reporting of sample size requirements and 2) sample size too small (<600) | 1) no, no reporting of requirements, and 2) yes, enough participants (>300) | 1) no, reporting of requirements is lacking, and 2) yes, enough participants (>300) |
| *1.2 Representativeness* | Representation of real-world heterogeneity and diversity, requiring 1) reporting of data collection and baseline characteristics, and 2) reporting where representation is missing | 1) yes, baseline variables in supplement, and 2) yes, they comment that their study is representative because of the lack of selection | 1) yes, report of how variables were measured and what the baseline characteristics were, and 2) no, because no discussion specifically on how well the general HFpEF population is represented | 1) yes, detailed report of data collection is referenced, as well as a baseline table for all characteristics, and 2) no, report of representation is lacking | 1) yes, elaborate explanation of data collection and reporting of baseline characteristics, 2) no, mention of generalizability or representation is missing | 1) yes, reporting of baseline characteristics and data collection, and 2) no, only ethnicity is mentioned as decreased representation, but other selection variables are not mentioned | 1) yes, reporting of data collection and baseline characteristics, and 2) yes, reported missing of representation and discussing the importance of using data from multiple countries | 1) yes, reporting of data collection, specifically very detailed report of the echocardiography, and baseline characteristics, 2) no, discussion on representation is missing |
| *1.3 Data quality* | 1) Description of missing data, 2) consideration of potential errors in measurements, and their underlying mechanisms, 3) reporting prospective or retrospective collection and 4) prospective collection is preferred | 1) yes, there is an overview of missing variables in the supplement, 2) no, because consideration of potential errors in measurements is not reported, and yes for 3 and 4 because they enrolled prospectively | 1) no, missingness is discussed in a non-transparent way as only some rates of missingness are reported but not specifically for each variable, 2) no, potential errors are not mentioned, 3) yes, reported, and 4) no, because retrospective | 1) no, description is lacking, and 2) no, reflection on potential errors is missing, and 3) no and 4) no, in methods its not mentioned its retrospective | 1) no, there is no mentioning of missing data and they only mention excluding patients with incomplete data, 2) no, there is no mentioning of potential errors in measurements, 3) yes and 4) yes, they mention that data collection was prospective | 1) yes, missing per variable is reported, 2) no consideration of potential errors discussed, 3) yes, and 4) yes, it is prospective | 1) no, description of missing data is lacking, 2) yes, describing how the echocardiograph can be wrong, 3) yes, reference to a paper where data collection is described, and 4) yes, it is prospective | 1) yes, missing for every variable is separately described, 2) no, mention of consideration of potential errors is missing, 3) yes and 4) yes, it is mentioned that the data were collected prospectively |
| *1.4 Data pre-processing* | Detailed description of 1) removing outliers, re-coding or transforming, standardization, and 2) imputation preferred over complete case analysis, and 3) detailed description of imputation of missing data (MAR, MCAR, MNAR) | 1) yes, they mention how standardized towards a mean 0 and a standard deviation of 1, 2) yes, they imputed using the SVDimpute function using regression with eigenvectors as predictors, 3) no, they do not discuss cause of missing data | 1) yes, description of handling of the data, 2) yes, imputation was used, 3) no, there was no description of details of the imputation process | 1) yes, report of standardization such as log-transformation to a z-score for some variables, 2) yes, using random forest imputation, and 3) yes, elaborate description of imputation process | 1) no, description of transforming variables is very limited to only a few variables, and no mentioning of outliers, re-coding, or standardization. 2) no, imputation is not mentioned, and 3) no, description of imputation or missing data is lacking'. | 1) no, data pre-processing very limited reported, 2) yes, imputation mentioned, and 3) and no, detailed description imputation lacking | 1) no mention of outliers, recoding, or standardization, 2) no mention of missing data handling, and 3) no description of imputation | 1) yes, mentions standardization according to Gower dissimilarity, 2) yes, imputation used, and 3) no, no detailed description available of the imputation process and assumptions of the missingness |
| **2. Development of the model** | |  |  |  |  |  |  |  |
| *2.1 Model selection and interpretability* | 1) Motivation for model selection clearly articulated including potential risks, 2) facilitating interpretability of model, 3) careful selection of number of clusters | 1) yes, they mention downsides of hierarchical clustering, 2) no, only a table and large heatmap are provided, and 3) yes, BIC was used | 1) no, motivation for use of LCA is missing, 2) yes, elaborate explanation of the possibilities and applications of this model, 3) yes, BIC used | 1) yes, elaborate explanation of multiple steps and checks and the reason behind them to get to the final model 2) no, clear visualisation of subgroups or in-depth description or discussion is missing, and 3) yes, BIC and Dunn index used | 1) yes, description of why k-medoids algorithm is a more robust alternative to k-means clustering, and the algorithm is explained. 2) no, visual aid to understand the cluster patterns is minimal and there is no explanation of the cluster characteristics, 3) no, description of how number of clusters was decided on is missing | 1) no, many analysis techniques used without properly discussing each of their advantages or disadvantages, and 2) no, using different rounds of clustering with different variables make interpretability very hard, and 3) yes, use of BIC | 1) no, motivation not mentioned, 2) yes, subgroups are explained although visual explanation is missing, except for a heatmap that shows how the biomarkers differ across the different phenogroups, and 3) yes, using LR-test, AIC, and BIC | 1) no, potential risks not mentioned, 2) yes, elaborate discussion on why clustering is needed in the field and comparison with outcomes from other clustering studies, and 3) no, mention of number of cluster selection is lacking |
| *2.2 Feature selection* | 1) Description of feature selection and feature meaning, 2) removal of highly correlated features, and 3) limiting number of features | 1) yes, they describe they filtered variables with high correlation and selected the variable that was most informative and had the least missingness, 2) yes, and 3) no, they used all 46 variables after removing the highly correlated variables | 1) yes, elaborate explanation of feature selection, 2) yes, correlation checked, 3) yes, reduced to 11 features | 1) no, explanation feature meaning, and rationale of initial selection is minimal, 2) yes, variables with a correlation of >0.6 are removed. 3) yes, Cohen's kappa is used to limit the number of variables from 61 to 20. | 1) no, there is no mentioning of a feature selection rationale or meaning of the features, 2) no, there is no testing of high correlation between variables, and 3) no, all variables available seem to have been selected | 1) no, meaning of features is not discussed, especially not the plasma proteins, and 2) yes, highly correlated features were removed, and 3) yes, some steps of limiting number of features were taking | 1) yes, they describe they chose clinical covariates that are widely available. 2) no, not described, and 3) yes, although they had many variables available, they chose to go with a limited subset of only 8 covariates | 1) no, no rationale for the selected features mentioned, 2) no mentioning of removal of highly correlated features, and 3) no, because they used a large amount (n=55) of features |
| *2.3 Internal validation* | 1) Report measures of internal validation such as reliability or stability of subgroups, 2) meaningfulness of found subgroups, and 3) comparable subgroup sizes with >5% of total number of participants in each subgroup | 1) no, because there is no testing or reporting on how stable or reliable subgroup assignment is, and 2) yes, subgroups characteristics are discussed in-depth, and 3) yes, comparable subgroup sizes (n=120-149) | 1) yes, probability of subgroup assignment is reported, 2) yes, in-depth discussion of characteristics of subgroups, 3) yes, all subgroups bigger than 5% of total participants | 1) no, report lacking reliability or stability, 2) no, very marginal discussion of meaningfulness of subgroups and mainly focused on outcome differences, 3) no, group 3 is almost four times as big as the other two subgroups. | 1) no, there is no mentioning of internal validation measures, 2) no, description of found clusters does not go beyond describing their key characteristics, and 3) yes, all subgroups contain more than 5% of the total study population | 1) no reliability or stability mentioned, and 2) no in-depth discussion of founds subgroups, and 3) no, subgroup sizes differ significantly with one subgroup containing less than 5% of the total number of participants (n=30-101) | 1) no mention of internal validation, 2) yes, good discussion on meaning of subgroups including possible aetiologies of the phenogroups according to their biomarkers, 3) yes, subgroups sizes are comparable (n=899-1329) | 1) no mention of reliability or stability of subgroups, 2) yes, discussion on found subgroups including how their aetiologies relate to current literature including literature on other clustering within HFpEF, 3) yes, comparable subgroup sizes |
| *2.4 Measures to reduce risk of overfitting* | 1) Ratio sample size and candidate features at least 30:1, and 2) penalizing model complexity | 1) no, sample size < 1380 (30*46), 2) yes, using AIC | 1) yes, >330 participants, 2) yes, using BIC | 1) no, less than 1830 participants (61*30), 2) yes, BIC used | 1) no, less than 30*20= 600 participants. 2) no, report of penalizing model complexity is missing. | 1) no, subgroup size allows for only 10 features, and they used 43, and 2) yes, use of BIC | 1) yes, more than 30*8 = 240 participants, and 2) yes, used BIC for #k selection and limited features | 1) no, less than 1650 participants (55*30), 2) no, no mention on how model complexity is limited |
| *2.5 Transparency modelling process* | 1) Reporting software packages and versioning, 2) publishing of the code for the complete model building pipeline, and 3) providing subgroup model algorithm for application | 1) no, lacking versioning report, and 2) no, no code published, and 3) also no model algorithm provided | 1) yes, versions reported, 2) no, code was not published, 3) yes, algorithm or probabilities provided | 1) yes, versioning mentioned, 2) no, no code is provided, and 3) no, algorithm for application is missing | 1) no, only R version is mentioned but no mentioning of the packages and their versioning. 2) no, code not provided, 3) no, algorithm not provided | 1) yes, reporting packages and their versioning, 2) no code published and, 3) no subgroup algorithm provided | 1) yes, versioning mentioned, 2) no, code was not published, and 3) no, subgroup algorithm not provided | 1) no, reports on versioning incomplete, 2) no code published, and 3) no subgroup algorithm |
| **3. Validation of the model** | |  |  |  |  |  |  |  |
| *3.1 External validation* | 1) Validation of model in different dataset, and 2) validation performed by independent researchers from other institutions or settings | 1) yes, validation in different subset of data, and 2) no, not performed by independent researchers or other institutions | 1) yes, validation in CHARM-PRESERVE, 2) no, performed by same researchers | 1) yes, external validation in other dataset, 2) no, same researchers | no validation | no validation | no external validation | no external validation |
| *3.2 Generalizability* | 1) Validate generalizability of the model using external data from different time period, place, or healthcare setting, 2) validate in dataset with sufficient sample size | 1) no, because validation cohort from same dataset, 2) no, because sample size validation was 100 which is less than the 300 required | 1) yes, small differences between dataset, and 2) yes, sample size big enough in the validation cohort | 1) yes, validation in different place, 2) no, sample size smaller than 600 (20*30) | no validation | no validation | no external validation | no external validation |

| **1. Preparation, collection and checking of the data** | | **Harada**[23] | **Stienen**[24] | **Gu**[25] | **Uijl**^18^ | **Casebeer**^19^ | **Woolley**^20^ |
| --- | --- | --- | --- | --- | --- | --- | --- |
| *1.1 Sample size* | 1) Reporting of sample size requirements and 2) at least 100 events or participants per outcome (subgroup) | 1) no, sample size requirements are not mentioned. 2) no, for initial clustering they used only 350 participants of the 483, and for 4 clusters they would need 400 | 1) no, sample size requirements not mentioned. 2) yes, more than 200 participants per subgroup | 1) no report of sample size requirement, 2) yes, enough participants (>300) | 1) no, reporting of requirements is lacking, and 2) yes, enough participants (>500) | 1) no, report is missing, and 2) yes, enough participants (>300) | 1) no, report is missing, 2) no, because model identified six subgroups and they have <600 participants |
| *1.2 Representativeness* | Representation of real-world heterogeneity and diversity, requiring 1) reporting of data collection and baseline characteristics, and 2) reporting where representation is missing | 1) Yes, elaborate explanation of how the data is collected and report of the baseline characteristics. 2) yes, explanation of how the inclusion criteria limit the generalizability of the sample, for example that obese patients might have been underrepresented because of the issues regarding imaging with those patients | 1) yes, elaborate description of protein biomarker measurements and baseline table provided. 2) yes, they mention that their HFpEF cohort consists of chronic and recently discharged HFpEF patients and that results can therefore not be extrapolated to more advanced HFpEF populations | 1) yes, baseline characteristics are reported, and 2) yes, they mention generalizability might be affected due to using participant from one centre. | 1) yes, report of when and how the data was collected, and which databases were linked, and 2) yes, they mention that they aimed to have good representation of the average HF patient that presents to the clinic | 1) yes, reporting of data collection and the corresponding ICD codes, and baseline characteristics, and 2) yes, decrease in generalizability is hypothesized as a result of using ICD codes for inclusion. | 1) no, overall baseline characteristics not reported, and 2) yes, discussed that cohort is mainly Caucasian |
| *1.3 Data quality* | 1) Description of missing data, 2) consideration of potential errors in measurements, and their underlying mechanisms, 3) reporting prospective or retrospective collection and 4) prospective collection is preferred | 1) no, no mentioning of missing data, 2) yes, some errors are discussed including the insufficiency for echocardiography to detect ATTRwt. 3) yes, they mention it is retrospective, 4) no, because retrospective | 1) yes, detailed description of missing. 2) yes, in the discussion they mention how variability of diastolic function makes HFpEF classification less reliable. 3) yes, they mention prospective. 4) yes, prospective | 1) no, no description of missing data or mentioning thereof 2,) no, potential errors are also not mentioned 3) yes reported and, 4) yes, prospectively collected | 1) yes, percentage of missing for each variable is reported, 2) yes, it is discussed that sometimes the indication for medication prescription is missing in the data, 3) yes, and 4) no, retrospective | 1) no, they discuss how they handled missing data but do not describe where the data was missing and how much, 2) yes, discussing issues of text-mining, 3) yes, reporting that data collection was retrospective, 4) no, retrospective | 1) no, very limited description of missing data, only mentioning how many patients had missing data, 2) no, no mentioning of potential errors, 3) no, report is lacking, and 4) no, it seems like data collection was retrospective |
| *1.4 Data pre-processing* | Detailed description of 1) removing outliers, re-coding or transforming, standardization, and 2) imputation preferred over complete case analysis, and 3) detailed description of imputation of missing data (MAR, MCAR, MNAR) | 1) yes, they mention that they standardize all variables using z-scores. 2) no, imputation is not mentioned. 3) no, no description of missing data or imputation present. | 1) yes, they describe how many variables were below lower limit of detection and that those were excluded from the analysis. 2) yes, imputation was used. 3) yes, they describe which parameters they used and that they used Rubin's rule for pooling. | 1) no, only standardization mentioned quickly but no explanation on how standardization was done, 2) no, missing not mentioned, and 3) no description of imputation | 1) yes, they describe how the variables are recoded for analysis, 2) no, complete case analysis used, 3) no, no imputation mentioned | 1) yes, description of transformation into dichotomous variables and removing variables with too low variance, 2) no, mention of imputation is lacking, and 3) no imputation mentioned | 1) yes, description of how proteomics values are standardized and transformed, 2) no, missing data handling barely mentioned, and 3) no, because no imputation mentioned |
| **2. Development of the model** | |  |  |  |  |  |  |
| *2.1 Model selection and interpretability* | 1) Motivation for model selection clearly articulated including potential risks, 2) facilitating interpretability of model, 3) careful selection of number of clusters | 1) yes, mechanisms of k-means clustering is explained and they mention that number of cluster selection is crucial for clustering performance, 2) yes, they not only explain how the clustering works but also visualize the model using principal components 3) yes, Hartigan's rule used for selection of number of clusters | 1) no, rationale behind model selection not mentioned. 2) yes, a visualization of the biomarker pathway is shown to understand the meaning of clustering using biomarkers. 3) yes, they used Clusterboot and Jaccard similarities to decide on model selection | 1) no, motivation not mentioned, 2) no, interpretation not mentioned, and 3) yes, BIC was used | 1) no, motivation for LCA not mentioned, 2) yes, model nicely visualized, 3) yes, elaborate explanation including use of BIC | 1) no, motivation not mentioned, 2) yes, subgroups discussed and visualized, 3) yes, objective analysis methods were used but not the BIC | 1) no, reason for model selection not mentioned, 2) yes, heatmap supports interpretability of the model, 3) yes, objective tool used for number of cluster selection |
| *2.2 Feature selection* | 1) Description of feature selection and feature meaning, 2) removal of highly correlated features, and 3) limiting number of features | 1) no, feature meaning not explained and there is no rationale mentioned for the selection of features. 2) no, taking into account correlation between features is not mentioned. 3) no, it seems like they included all initial features | 1) yes, a figure shows the cellular processes that the biomarkers are associated with. 2) yes, biomarkers with a correlation of >0.8 were removed. 3) yes, they reduced number of features using correlation and missingness | 1) no, description is lacking, 2) no mentioning of removal of highly correlated features, and 3) yes, number of features are limited to a selection of 11 variables | 1) yes, rationale for feature selection mentioned, 2) no, no mentioning of correlation, and 3) yes, number of features was limited to 10 'most important' variables according to clinician input and pragmatic reasons | 1) yes, feature selection explained and rationalized, 2) yes, variables with high correlation were removed 3) yes, trying to simplify the model through variable selection, using an unsupervised approach for variables selection | 1) yes, described PCA for feature reduction and the role of each component, 2) yes, PCA deals with this, 3) yes, features are reduced to 59 components |
| *2.3 Internal validation* | 1) Report measures of internal validation such as reliability or stability of subgroups, 2) meaningfulness of found subgroups, and 3) comparable subgroup sizes with >5% of total number of participants in each subgroup | 1) yes, internal validation was performed using a subset of the original cohort to check robustness of found subgroups. 2) yes, in-depth discussion on the implications of found subgroups. 3) yes, subgroup sizes do not go below 5% of the total (n = 44-157) | 1) yes, clusterboot was used to determine cluster stability. 2) yes, they describe that they find processes related to immune system, signal transduction cascades, and cell interactions and metabolism associated with cluster assignment. 3) yes, all cluster sizes exceed 5% of total sample size (n = 163 - 229) | 1) yes, internal validation consisted of assigning a separate validation cohort according to the previously developed cluster algorithm and compare subgroup outcomes, 2) yes, discussion on possible applications of found subgroups and how they relate to other studies, and 3) yes, subgroup sizes all more than 5% of total sample size (N= 240 - 423) | 1) yes, probabilities of cluster assignment are reported which is a measure of cluster stability, 2) yes, in-depth discussion of meaning of findings including the underlying aetiologies of found subgroups, 3) yes, all subgroups contain more than 5% of the total sample size (n = 264 - 721) | 1) no, reliability or stability of subgroups not discussed, 2) yes, meaning of found subgroups discussed and compared to previous clustering studies, 3) yes, all subgroup sizes bigger than 5% of the total sample size (N = 444 - 557) | 1) no, reliability or stability of subgroups not mentioned, 2) yes, in-depth discussion on found subgroups and their implications, 3) no, one subgroup with N=2 and one with N=3 |
| *2.4 Measures to reduce risk of overfitting* | 1) Ratio sample size and candidate features at least 30:1, and 2) penalizing model complexity | 1) no, less than 37*30= 1110 participants, 2) yes, Hartigans rule was used to select number of clusters | 1) no, less than 30*349=10470 patients. 2) no, statistics used do not penalize model complexity | 1) yes, more than 330 participants (30*11), 2) yes, BIC used | 1) yes, >300 participants (30*10), 2) yes, use of BIC | 1) yes, >360 participants (30*12), and 2) yes, pseudo-F statistic used to determine number of clusters | 1) no, less than 1770 participants in the sample (30*59), 2) no, don't see measures that penalize model complexity like BIC |
| *2.5 Transparency modelling process* | 1) Reporting software packages and versioning, 2) publishing of the code for the complete model building pipeline, and 3) providing subgroup model algorithm for application | 1) yes, packages mentioned including their version. 2) No, code not published. 3) yes, the coordinates of the cluster centroids are provided which can be used for application of the model | 1) no, versioning is lacking. 2) no, code not published. 3) no, no algorithm is provided | 1) no, versioning incomplete, 2) no code published, 3) no, no algorithm provided | 1) no, versioning incomplete, 2) no publishing of code, 3) yes, subgroup probabilities published that can be used to apply the model to other datasets as well. | 1) yes, versioning mentioned, 2) no, code not published, 3) no, algorithm not published | 1) no, versioning is lacking, 2) no, code is not published, and 3) no, subgroup model algorithm not provided |
| **3. Validation of the model** | |  |  |  |  |  |  |
| *3.1 External validation* | 1) Validation of model in different dataset, and 2) validation performed by independent researchers from other institutions or settings | no external validation | no external validation | no external validation | 1) yes, external validation of subgroup probabilities and subgroup outcomes in CHECK-HF, 2) no, performed by same researcher | no external validation | no external validation |
| *3.2 Generalizability* | 1) Validate generalizability of the model using external data from different time period, place, or healthcare setting, 2) validate in dataset with sufficient sample size | no external validation | no external validation | no external validation | 1) yes, different country, 2) yes, sufficient sample size | no external validation | no external validation |

| **1. Preparation, collection and checking of the data** | | **Nouraei**^21^ | **Perry**^22^ | **Fayol^23^** | **Murray^24^** | **Choy^25^** | **Banerjee^26^** |
| --- | --- | --- | --- | --- | --- | --- | --- |
| *1.1 Sample size* | 1) Reporting of sample size requirements and 2) at least 100 events or participants per outcome (subgroup) | 1) no reporting of sample size requirements, and 2) no, way less than 600 participants | 1) no, reporting requirements is missing, 2) yes, enough participants (>700) | 1) no, sample size requirements not mentioned. 2) yes, enough participants (>300) | 1) No, sample size requirements not mentioned. 2) Yes, enough participants (>400) | 1) no, sample size requirements are not discussed. However, in the discussion they mention that this study was underpowered to find an effect of the drug. 2) Yes, enough participants (>300) | 1) no, sample size requirements are not reported. 2) yes, enough participants included (>500) |
| *1.2 Representativeness* | Representation of real-world heterogeneity and diversity, requiring 1) reporting of data collection and baseline characteristics, and 2) reporting where representation is missing | 1) yes, data collection and baseline characteristics presented, and 2) no, no discussion on representation | 1) yes, reporting baseline and data collection, and 2) yes, mentioning that their population do not represent all HFrecEF patients | 1) Yes, elaborate explanation of how they retrieved patient information through text mining and how multiple cardiologists reviewed the medical records. Also baseline characteristics are reported. 2) Yes, they checked whether the baseline characteristics are in line with a real world unselected cohort of HFpEF patients | 1) Yes, there is a reference to an article where data collection is described, and there is also a baseline table present. 2) Yes, it is discussed that representation of patients with valvular disease and renal disease is lacking, as these comorbidities were used as exclusion criteria in the ASCEND-HF study. | 1) yes, baseline table is reported and process of data collection is explained. 2) Yes, discussion on how the USA and Russia/Georgia cohorts in the TOPCAT study differed significantly and represent very different populations. | 1) yes, they describe how the datasets are created and where the data linkage took place, and they also report baseline characteristics of the subgroup from both datasets. 2) yes, they discuss that a limitation for representation is that the datasets are limited to one country, but also that the fact that the data is an EHR helps with representation of the general HF population |
| *1.3 Data quality* | 1) Description of missing data, 2) consideration of potential errors in measurements, and their underlying mechanisms, 3) reporting prospective or retrospective collection and 4) prospective collection is preferred | 1) yes, missing data described, 2) no, discussion on erroneous measurement is missing, and 3) yes, mentioned, and 4) no, retrospective | 1) no, description of missing data is lacking, 2) yes, LVEF inaccuracy is mentioned, 3) yes, mentioned that study is retrospective, and 4) no, retrospective | 1) No, missing is only shortly mentioned in the limitations but there is no description of how much data was missing and in which variables the missing was present. 2) No, it is not mentioned where potential errors could be introduced. 3) Yes, they report that data was collected retrospectively, and 4) no, not prospective. | 1) No, only short description that variables with 'significant' missing data were excluded, but no further details are described regarding missingness. 2) No, potential errors introduced in the measurement process are not mentioned, 3) No, it is not mentioned in the paper whether data collection was retrospective or prospective. and 4) Yes, reading the study design (a trial setting) it seems most likely that data collection was prospective | 1) No, only shortly mentioned that variables with a certain fraction missing will be excluded, but a detailed description of the missing is not present. 2) No, errors within the measurements are not discussed. 3) No, it is not mentioned whether data was collected retro- or prospectively. 4) Yes, reading the study design and considering that this is trial data, the data seems to be collected prospectively. | 1) no, limited report of missing data while they only describe that they excluded variables that exceeded a threshold of percentage of missingness, 2) no, they do discuss some disadvantages of using EHR data such as that NT-pro-BNP measurements often are lacking, but do not discuss whether there could be errors in the data that was available. 3) Yes and 4) yes, they mention the data is recorded prospectively for both datasets. |
| *1.4 Data pre-processing* | Detailed description of 1) removing outliers, re-coding or transforming, standardization, and 2) imputation preferred over complete case analysis, and 3) detailed description of imputation of missing data (MAR, MCAR, MNAR) | 1) yes, mentioning of Gowers distance, 2) yes, missing data imputed, and 3) no, detailed description on imputation is missing. | 1) yes, mentioning of Gowers distance to recode and standardize variables, 2) no, report of missing data is lacking, 3) no, again there is no report of missing data | 1) Yes, using Gower metric to transform and standardize the variables. 2) no, handling of missing data is not mentioned. 3) No, a description of missing data is lacking. | 1) Yes, description of standardization of the variables to a mean of zero and standard deviation of one. Also, variables were clustered, but an explanation of the techniques used is missing. 2) No, variables with missing were just excluded. 3) No, no imputation was used. | 1) no, there is no mentioning of these data pre-processing steps, 2) no, only missing handling technique they discussed is excluding variables with a large fraction missing. 3) no, report on missing and the assumptions underlying them is lacking. | 1) no, there is no description of outliers or standardization of the data. 2) yes, imputation using PCA and MCA. 3) No, there is no detailed description of the missingness in the data and the assumptions linked to it. |
| **2. Development of the model** | |  |  |  |  |  |  |
| *2.1 Model selection and interpretability* | 1) Motivation for model selection clearly articulated including potential risks, 2) facilitating interpretability of model, 3) careful selection of number of clusters | 1) no, potential risks not mentioned, and 2) yes, visual explanation of subgroups and in-depth discussion of what they consist of, 3) yes, silhouette tool is used | 1) no, potential risks not mentioned, 2) no, hard to get an overview of all cluster characteristics and how they are modelled, 3) yes, silhouette method used. | 1) No, rationale and risks of chosen model are not discussed. 2) yes, a visualization of the hierarchical clustering and the distribution of variables is shown which helps to interpret the outcomes of the model. 3) Yes, silhouette method was used for number of cluster selection. | 1) Yes, they selected hierarchical clustering to work around having a fixed number of clusters. 2) No, visual aid to understand the model, and also no explanation of how unsupervised clustering exactly works. Only the subgroup characteristics and their implications are discussed. 3) yes, three statistics were combined to find the optimum number of clusters | 1) yes, they explain the swap-stepwise algorithm and its pitfalls. 2) yes, colour-coding and mentioning the key characteristics of the phenogroups throughout the paper make all tables and figures easy to understand. 3) yes, multiple measures including AIC used to determine optimal number of clusters. | 1) no, because although they used four different ML models they do not explain the risks or advantages of each approach. 2) yes, there is visual aid to understand what the different clusters look like and also an online shiny app to easily apply the algorithm yourself. 3) yes, they used the silhouette method to define ideal number of clusters. |
| *2.2 Feature selection* | 1) Description of feature selection and feature meaning, 2) removal of highly correlated features, and 3) limiting number of features | 1) no, only rationale mentioned was about correlation, 2) yes, correlation of >0.6 removed, 3) no, it is unclear which variables are used in the model | 1) yes, rationale mentioned, 2) no, correlation not mentioned, 3) yes, small number selected | 1) No, it is not explained how they reached the final selection of variables. 2) No, correlation is not mentioned, and it is not clear whether correlated features were excluded. 3) Yes, it seems that 11 features is a selection of all variables that were available. | 1) No, there was no selection of variables. 2) yes, there was clustering of variables which could mean that highly correlating variables were clustered. 3) No, it seems that all available features (39) were included in the analysis | 1) Yes, they describe they used a data-science driven approach for variable selection, including explanation of this algorithm. 2) Yes, assuming that the swap-step algorithm will not include highly correlated variables. 3) Yes, they reduced features to ten final variables. | 1) Yes, they used a ML approach to define most informative and useful variables, reducing the number of variables from 645 to 87 using Random Forest supervised classification with 1-year mortality. 2) Yes, Random Forest will filter highly correlated variables. 3) Yes, they reduced the number of variables by 7-fold. |
| *2.3 Internal validation* | 1) Report measures of internal validation such as reliability or stability of subgroups, 2) meaningfulness of found subgroups, and 3) comparable subgroup sizes with >5% of total number of participants in each subgroup | 1) no, as they do talk about internal validation but are not measuring it correctly (they test for differences in clinical characteristics), 2) yes, they talk about subgroup characteristics and whether they overlap with other studies, and 3) yes, comparable subgroup sizes | 1) no, discussion on internal validation is very minimal, 2) yes, meaning of found subgroups discussed in-depth, 3) no, huge differences in subgroup sizes | 1) No, there is no form of internal validation reported. There is no mentioning of reliability, validity, or robustness of the model. 2) Yes, they discuss how the clusters relate to their aetiologies and how both could be used in the clinic to handle the heterogeneity of HFpEF. 3) Yes, all subgroups contained more than 5% of the total sample size (n = 125 - 510) | 1) Yes, they compared found subgroups with the subgroups from the algorithm that was provided by Kao *et al.* 2) Yes, in-depth discussion of the cluster characteristics and their underlying aetiologies. 3) Yes, all clusters contained more than 5% of the total sample size (n = 171 - 273) | 1) Yes, a sensitivity analysis was performed to investigate the impact of excluding the non-US part of the dataset. 2) yes, they discuss possible underlying causes for found subgroup characteristics. 3) yes, all subgroups contained more than 5% of total sample size (n = 390 - 737) | 1) yes, they compared the performance of different clustering approaches to ensure internal validity. 2) yes, they discuss the expected underlying mechanisms for the different subgroups. 3) yes, all subgroup sizes bigger than 5% of total sample size (n = 28,617 - 105,610) |
| *2.4 Measures to reduce risk of overfitting* | 1) Ratio sample size and candidate features at least 30:1, and 2) penalizing model complexity | 1) no, number of candidate features unknown but also very small sample size, 2) no, no mentioning of using tools such as BIC that penalize model complexity | 1) yes, >330 (30*11) participants, 2) no, could not find objective tools that penalize model complexity like BIC | 1) yes, more than (11*30) 330 participants. 2) Yes, Ward's method used for the hierarchical clustering | 1) No, there are less than 30*39 = 1160 participants. 2) Yes, multiple statistics used to decide optimum number of clusters. | 1) yes, more than 30*10 = 300 participants. But before variable reduction through the swap-step algorithm they had too little participants. 2) Yes, AIC was used. | 1) Yes, sample size is bigger than 87*30=2610 patients. 2) Yes, silhouette method penalizes model complexity. |
| *2.5 Transparency modelling process* | 1) Reporting software packages and versioning, 2) publishing of the code for the complete model building pipeline, and 3) providing subgroup model algorithm for application | 1) yes, versioning mentioned, 2) no, code not published, and 3) no, algorithm not published | 1) yes, packages and versioning mentioned, 2) no code published, and 3) no algorithm published | 1) No, names and versioning of all packages is missing. 2) no code published. 3) No, model algorithm not provided. | 1) no, there are no packages or versioning mentioned. 2) no code is provided. 3) no, subgroup model algorithm is not shared. | 1) no, versioning is missing. 2) no, code is not published. 3) no, subgroup algorithm not supplied. | 1) No, only version of R and Python is mentioned but not the used packages and their version. 2) No, no code provided. 3) Yes, they provide a shiny app where you can fill in the patient details to get a cluster assignment and a risk prediction, however you cannot take this app and apply it on large scale. |
| **3. Validation of the model** | |  |  |  |  |  |  |
| *3.1 External validation* | 1) Validation of model in different dataset, and 2) validation performed by independent researchers from other institutions or settings | no external validation | no external validation | no external validation | no external validation | no external validation | 1) Yes, performed in CPRD. 2) No, validation seems to be performed by the same researchers |
| *3.2 Generalizability* | 1) Validate generalizability of the model using external data from different time period, place, or healthcare setting, 2) validate in dataset with sufficient sample size | no external validation | no external validation | no external validation | no external validation | no external validation | 1) No, both datasets are from primary care within the UK, and even have overlap regarding some patients. 2) Yes, validation cohort is 188,799 patients. |

| **1. Preparation, collection and checking of the data** | | **Kao^27^** | **Ahmad^28^** | **Ferreira^29^** | **Tromp^30^** | **Karwath^31^** | **Bouali^32^** | **de Lange^33^** |
| --- | --- | --- | --- | --- | --- | --- | --- | --- |
| *1.1 Sample size* | 1) Reporting of sample size requirements and 2) at least 100 events or participants per outcome (subgroup) | 1) no, sample size requirements not mentioned. 2) yes, more than 600 patients. | 1) yes, they mention that they could not identify more than 4 clusters because of power considerations. 2) yes, more than 400 patients included | 1) no, sample size requirements not mentioned. 2) yes, more than 400 participants. | 1) no, sample size requirements not mentioned. 2) yes, more than 600 patients. | 1) no, sample size requirements not mentioned. 2) yes, more than 1100 participants | 1) no, sample size requirements not mentioned. 2) no, less than 200 patients. | 1) yes, they discuss that sample size was limited. 2) no, less than 300 patients |
| *1.2 Representativeness* | Representation of real-world heterogeneity and diversity, requiring 1) reporting of data collection and baseline characteristics, and 2) reporting where representation is missing | 1) yes, baseline characteristics reported, and data collection can be found in references of corresponding trials. 2) yes, they discuss how the inclusion and exclusion criteria of the models developed from BEST are problematic for generalizability. | 1) yes, baseline characteristics reported, and data collection described. 2) yes, they discuss that current phenogroups are highly dependent on chosen variables and the dataset that is used | 1) yes, reference to paper with detailed report of data collection, 2) yes, they describe which populations the two datasets represent. | 1) yes, all stages of data collection are explained, and baseline table is provided in the supplements. 2) yes, they mention that almost all patients are Caucasian and that this might limit their generalizability. | 1) yes, baseline characteristics are reported. Also, and the included trials are described as well as how the data was extracted. 2) no, they only mention that there is a broad representation of trials across the cluster but do not further discuss representation or generalizability. | 1) yes, baseline table and detailed description of measurement collection is present. 2) no, discussion on generalizability and representation is missing. | 1) yes, detailed description of data collection and a baseline table are provided. 2) yes, limitation regarding generalizability discussed, stating that results might not be generalizable to HF patients with for example advanced COPD or renal failure |
| *1.3 Data quality* | 1) Description of missing data, 2) consideration of potential errors in measurements, and their underlying mechanisms, 3) reporting prospective or retrospective collection and 4) prospective collection is preferred | 1) no, missingness is not mentioned. 2) no, potential errors not discussed. 3) yes, they mention retrospective analysis. 4) no, retrospective | 1) no, detailed description on missingness pattern is lacking. 2) no, potential errors in measurements not described. 3) no, it is not described whether this study was performed retrospectively or prospectively. 4) yes, considering that trial data is used for this paper, it is most likely that data was collected prospectively. | 1) yes, they described which variables had high proportion of missing how this can have influenced the analysis. 2) yes, they discuss how the haemoglobin levels, renal function, and hyperkalaemia levels might have been influenced by contrast agents. 3) yes, they mention retrospective, and 4) no, retrospective. | 1) no, they only mention how many patients they had to exclude for complete case analysis. 2) no, they do not discuss where there could be errors introduced in the measurements. 3) yes, they refer to the original paper for the BIOSTAT-CHF dataset where they mention it was prospectively collected. 4) yes, prospective. | 1) yes, in the description of Figure 1 there is a detailed description of all missing in the data. 2) yes, they mention they might have missed paroxysmal atrial fibrillation and that they have no information on the duration or burden of atrial fibrillation. 3) no, it is not mentioned whether it is prospective or retrospective. 4) yes, trial data is usually collected prospectively, and this dataset consists of trial data | 1) yes, detailed description of missing per variable. 2) no, potential errors of measurements are not discussed. 3) yes, they mention prospective. 4) yes, prospective. | 1) no, they only shortly mention there is missingness, but they do not describe in which variables. 2) no, they do not discuss potential errors in their measurements. 3) yes, they mention their research is prospective. 4) yes, prospective |
| *1.4 Data pre-processing* | Detailed description of 1) removing outliers, re-coding or transforming, standardization, and 2) imputation preferred over complete case analysis, and 3) detailed description of imputation of missing data (MAR, MCAR, MNAR) | 1) no, description of pre-processing of the data is missing, 2) no, only for percent lymphocytes they use a fixed value to fill all missingness, 3) no, assumptions of missingness not discussed. | 1) yes, they standardized the 13 variable score to have a mean zero and a standard deviation of 1. 2) no, they used complete case analysis. 3) no, they did not use imputation. | 1) yes, they describe recoding and the rationale behind it, 2) no, they used complete case analysis, and 3) no, they used complete case analysis. | 1) yes, expression data is log2 normalized. 2) no, they used complete case analysis. 3) no, complete case analysis | 1) no, although they mention that they pre-processed the data there is no description of how they handled outliers, recoding or standardization. 2) no, they performed complete case analysis. 3) no, complete case analysis. | 1) yes, Gower's distance is used for standardization. 2) yes, missing values are imputed. 3) no, they only mention which package was used but do not mention assumptions regarding the missingness. | 1) yes, they performed mixed modelling to compute individual intercept and slopes of the temporal biomarker trajectories, and subsequently performed winsorization and standardization. 2) yes, they used single imputation. 3) no, they do not discuss details of the imputation or the assumptions of the missingness |
| **2. Development of the model** | |  |  |  |  |  |  |  |
| *2.1 Model selection and interpretability* | 1) Motivation for model selection clearly articulated including potential risks, 2) facilitating interpretability of model, 3) careful selection of number of clusters | 1) no, rationale for selection of algorithm not discussed. They do discuss why LCA is better than 'traditional' regression, but this does not justify why they chose specifically LCA as clustering algorithm. 2) yes, several tables and figures help understand the differences between the subgroups. 3) yes, BIC used. | 1) no, they describe the advantages of clustering in general but do not mention why they decided to use hierarchical clustering. 2) yes, visual aid to understand how the different clusters compare regarding outcomes and what their characteristics are. 3) yes, they used War's minimum variance method to decide on the number of clusters. | 1) no, rationale for model selection not mentioned. 2) yes, visual aid to understand the subgroup characteristics, and also figures that show the differences in outcomes between the subgroups. 3) yes, BIC used. | 1) yes, they explain they used different models on both the development as well as the validation data and chose the model that validated the best. 2) yes, they provide heatmaps and a summarizing figure in the supplements which help to understand the models. 3) yes, they used Nblust for optimal number of clusters. | 1) yes, they discuss the risk of artificial intelligence but also their advantages and how they have tried to make the models easier to interpret. 2) yes, they created figures that show the different characteristics of the subgroups and their different responses to beta-blockers. 3) yes, they used the Gap statistic to decide on the number of clusters. | 1) no, motivation for hierarchical clustering not mentioned. 2) no, there is no visual aid to support the findings from the modelling. But there is discussion of the underlying pathologies of the clusters. 3) yes, several statistics were used to determine optimal number of clusters. | 1) no, motivation is not mentioned. 2) yes, visual aid of the biomarker patterns helps understand how the model has clustered the patients. 3) yes, Nbclust package used to determine optimal number of clusters |
| *2.2 Feature selection* | 1) Description of feature selection and feature meaning, 2) removal of highly correlated features, and 3) limiting number of features | 1) yes, they perform two separate LCAs with different variables to reach different goals and explain the rationale behind it. 2) no, correlation not mentioned. 3) yes, they used a sub selection of the available variables | 1) yes, using PCA for dimensionality reduction. 2) yes, PCA decreases number of correlated features, 3) yes, they actively tried to bring down the dimensionality of the 45 initial variables. | 1) no, rationale for model selection not mentioned2) yes, visual aid to understand the subgroup characteristics, Feature also figures that show the differences se outcomes between the subgroups. and 3) yes, BIC used. | 1) yes, they describe that they used a heart disease panel for their biomarker selection. 2) yes, PCA will decrease number of highly correlated features. 3) yes, they performed PCA to decrease number of variables. | 1) yes, they selected a list of common variables available at baseline that are known to be relevant to outcomes in patients with HFrEF. 2) no, there is no description of correlation between variables and the removal thereof. 3)yes, they decided on using only six features. | 1) no, there is no description of why they selected current variables and how they did that. 2) yes, Gowers distance corrects for multicollinearity. 3) no, it seems like they included all available variables, or at least a description of limiting the number of variables is missing. | 1) no, they have no description of making a selection all variables available. 2) no, there is no description of removing highly correlated variables. 3) no, they included 92 variables |
| *2.3 Internal validation* | 1) Report measures of internal validation such as reliability or stability of subgroups, 2) meaningfulness of found subgroups, and 3) comparable subgroup sizes with >5% of total number of participants in each subgroup | 1) yes, they perform leave-one-out-cross-validation on the c-indices. 2) yes, they explain how the developed models can be used in a clinical setting and how they compared to current tools. 3) yes, all subgroups exceed 5% of total sample size (n = 87 - 317) | 1) yes, they investigated cluster stability using a bootstrap resampling method and applying the original clustering algorithm, subsequently comparing the hierarchical clustering with the Bk statistic. 2) Yes, they discuss implications of finding these clusters, 3) yes, all clusters were bigger than 5% of total sample size (n = 246 - 773) | 1) yes, in the supplements the cluster probabilities are reported which are a measure of cluster stability 2) yes, they explain how found clusters are hypothesis generating. 3) yes, all subgroups exceed the 5% of complete sample size threshold. | 1) no, interval validation measures are not mentioned. 2) yes, they discuss implications of their found subgroups including hypothesizing which subgroups benefit from which treatments. 3) no, one group contains less than 5% of the total sample size (n = 80 - 435) | 1) Yes. the robustness of the approach was confirmed using repeated clustering (k=100) of random subsets of the data (bootstrapping), comparing the resulting clustering against random cluster assignments. 2) yes, they mostly discuss the drug response of the subgroups and whether clustering can pose a solution in treatment research. 3) no, one sinus rhythm subgroup and all atrial fibrillation subgroups were smaller than 5% of the total sample size | 1) no, measures of internal validation not mentioned. 2) yes, they discuss the underlying biological mechanisms of the clusters and why one cluster responds better to treatment than the other. 3) yes, both subgroups exceed the 5% of the total sample size (n = 45- 63) | 1) yes, they performed resampling (including bootstrapping and noise replacement). 2) yes, explanation of the pathologies of the clusters. 3) yes, all subgroups have more than 5% of total sample size (n = 78 - 93) |
| *2.4 Measures to reduce risk of overfitting* | 1) Ratio sample size and candidate features at least 30:1, and 2) penalizing model complexity | 1) yes, more than 7*30=210 patients, 2) yes, BIC penalizes model complexity | 1) yes, more than 13*30 = 390 patients, 2) yes, Wards minimum variance penalizes model complexity. | 1) yes, more than 18*30=540 patients, 2) yes, BIC penalizes model complexity. | 1) no, less than 91*30 = 2730 patients. 2) yes, using Wards minimum variance for number of cluster selection | 1) yes, more than 30*6= 180 participants. 2) yes, Gap statistic penalizes model complexity. | 1) no, less than 30*17=510 patients. 2) yes, three different statistics used to determine optimal cluster number | 1) no, less than 92*30 = 2760 patients. 2) yes, nbclust penalizes model complexity. |
| *2.5 Transparency modelling process* | 1) Reporting software packages and versioning, 2) publishing of the code for the complete model building pipeline, and 3) providing subgroup model algorithm for application | 1) no, packages are mentioned but versioning is missing. 2) no, code is not provided. 3) no, algorithm for application is not provided | 1) yes, packages with their versioning are mentioned, 2) no, code not published, and 3) no, algorithm not provided. | 1) yes, packages and their versioning are reported. 2) no, code is not published. 3) yes, subgroup membership probabilities are provided. | 1) no, packages mentioned but versioning is missing. 2) no code is not provided. 3) no, algorithm is not provided. | 1) no, packages and software not mentioned. 2) no, code not provided. 3) no, subgroup model algorithm not provided. | 1) no, packages are mentioned but versioning is not complete. 2) no, code is not published. 3) no, model algorithm not provided. | 1) no, packages mentioned but versioning is lacking. 2) no, code is missing. 3) no, algorithm not provided. |
| **3. Validation of the model** | |  |  |  |  |  |  |  |
| *3.1 External validation* | 1) Validation of model in different dataset, and 2) validation performed by independent researchers from other institutions or settings | 1) yes, validation performed in different dataset. 2) no, same researchers. | no external validation | 1) yes, EPHESUS used for external validation of the subgroup outcomes. 2) no, same researchers, | 1) yes, they used a Scottish multi-centre dataset. 2) no, it is not mentioned that an independent researcher performed validation. | no external validation | no external validation | no external validation |
| *3.2 Generalizability* | 1) Validate generalizability of the model using using external data from different time period, place, or healthcare setting, 2) validate in dataset with sufficient sample size | 1) yes, different patient group and different time period, 2) no, sample size is smaller than 600 patients. | no external validation | 1) yes, EPHESUS contains different type of HF patients. 2) yes, more than 400 patients in the EPHESUS trial. | 1) yes, different place and different characteristics of HF patients. 2) yes, validation dataset was more than 600 patients. | no external validation | no external validation | no external validation |

| **1. Preparation, collection and checking of the data** | | **Ahmad^34^** | **Tromp^35^** | **Nagamine^36^** | **Gevaert^37^** | **Gulea^38^** | **Uszko-Lencer^39^** | **Zheng^40^** | **Zhou^41^** |
| --- | --- | --- | --- | --- | --- | --- | --- | --- | --- |
| *1.1 Sample size* | 1) Reporting of sample size requirements and 2) at least 100 events or participants per outcome (subgroup) | 1) no, sample size requirements not mentioned. 2) yes, more than 400 participants | 1) no, sample size requirements not mentioned. 2) yes, more than 500 patients. | 1) no, sample size requirements are not mentioned. 2) yes, more than 1500 patients. | 1) no, sample size requirements not mentioned. 2) yes, more than 600 patients. | 1) no, sample size requirements not mentioned, 2) yes, more than 500 patients | 1) no, they only mention their study consisted of a 'considerable number' of HF patients but do not discuss sample size requirements any further. 2) yes, more than 500 patients. | 1) no, they did not mention sample size requirements. 2) yes, more than 400 patients | 1) no, sample size requirements are not mentioned. 2) yes, more than 500 participants |
| *1.2 Representativeness* | Representation of real-world heterogeneity and diversity, requiring 1) reporting of data collection and baseline characteristics, and 2) reporting where representation is missing | 1) yes, it is explained how the most important variables were measured, for example that revascularization was defined as history of coronary artery bypass surgery or percutaneous coronary intervention. Also, a baseline table is present. 2) no, they mention that they only used Swedish patients but instead of arguing that this decreases generalizability towards other HF patients they state that this has decreased unmeasured confounders. | 1) yes, a reference for the report of the data collection is given, as well as a baseline table. 2) yes, they discuss that there is sparse data on heart failure within Asian countries and therefore it is hard to check whether current dataset is representative of general heart failure patients in Asia. | 1) yes, description how data was collected using text mining and baseline characteristics are reported. 2) no, generalizability or proper representation of HF patients is not discussed, despite that this study only includes Russian participants. | 1) yes, they explain how variables were extracted from registries and administrative databases, and baseline characteristics are reported. 2) yes, discussed that the findings might not be generalizable to other populations, but that all LVEF categories were represented in all found clusters | 1) yes, description of which patients were included and excluded, and how the data for the dataset was collected. Also, a baseline table is present. 2) no, they mention that previous research often uses trial data which is not a good representation of all HF patients, and they argue that using a population sample like they did with clinical variables gives a good representation of the people living in the US. So, they do not mention where representation is missing. | 1) yes, they describe how the different features are measured and provide a baseline table. 2) yes, they discuss that this dataset only represents a part of the total HF population and therefore results cannot be extrapolated to other groups. | 1) no, reporting of data collection is very limited. Baseline table is available. 2) yes, they mention that only including two hospitals may have led to selection bias. | 1) yes, they describe how the data was collected as they retrieved patient data from a single centre electronic health record, and baseline age and sex are mentioned. 2) yes, they discuss that their results are only applicable on Japanese population and that generalizability is less because they used data from only one centre. |
| *1.3 Data quality* | 1) Description of missing data, 2) consideration of potential errors in measurements, and their underlying mechanisms, 3) reporting prospective or retrospective collection and 4) prospective collection is preferred | 1) No, besides mentioning they excluded variables with more than 20% missing there is no detailed description on the missingness of the data. 2) no, it is not mentioned where there could be errors in the measurements. 3) no, it is not mentioned whether the data was collected retrospectively or prospectively. 4) yes, when looking into other clustering studies that used the SwedeHF dataset it becomes apparent that data was collected prospectively. | 1) no, missingness is not described. 2) yes, they discuss that there could be potential bias in site selection and willingness of patients to participate because of the geography of the dataset. 3) yes, they report prospective. 4) yes, prospective. | 1) no, there is no description of missing data. 2) no, although they do mention that using text mining in EHR can be challenging, there is no description or evaluation whether errors could have been introduced into their own data. 3) no, it is not specifically mentioned whether the data was collected retrospectively or prospectively. 4) yes, reading the dataset description it seems that the data was collected prospectively. | 1) no, besides mentioning that most variables had less than 3% missing, there is no further description of where exactly this missing happened. 2) yes, they discuss that event rates can have been biased by adherence to medical therapy and quality of care. 3) no, not reported whether data collection was prospective or retrospective. 4) yes, considering that data is from a trial, it is probably collected prospectively. | 1) yes, they mention the percentage of missing for a selection of variables. 2) yes, they mention that using administrative data can lead to misclassification and measurement error. 3) yes, they report retrospective data collection. 4) no, retrospective | 1) yes, detailed description of this missingness of each variable in the text below the tables. 2) no, potential errors in the measurements not mentioned. 3) yes, they mention retrospective. 4) no, retrospective. | 1) no, there is no description of how much missing there is exactly in each variable. 2) no, potential errors that could be introduced during measurement of the variables is not mentioned. 3) yes, prospective. 4) yes, prospective | 1) no, they only mention they exclude variables with more than 20% missing but do not elaborate where this missingness is. 2) no, they do not discuss possible errors in the measurements. 3) yes, they mention retrospective. 4) no, retrospective. |
| *1.4 Data pre-processing* | Detailed description of 1) removing outliers, re-coding or transforming, standardization, and 2) imputation preferred over complete case analysis, and 3) detailed description of imputation of missing data (MAR, MCAR, MNAR) | 1) yes, there is a description of how the most important variables are recoded. 2) yes, they used mean or most common value imputation. 3) no, the assumptions regarding the missingness are not discussed. | 1) yes, they describe how they categorized all continuous variables and why they used specific thresholds. 2) no, poLCA package uses complete case analysis for the analysis. 3) no, missingness is not discussed. | 1) yes, in-depth description of how data mining works and how the variables are created. 2) no, imputation not mentioned. 3) no, missingness is not discussed. | 1) yes, distances between variables are standardized using the Gowers distance. 2) no, imputation not mentioned. 3) no, there is no report on the assumptions of the missingness. | 1) no, how data is pre-processed regarding outliers, re-coding, transforming, or standardization is not mentioned. 2) no, they performed complete case analysis. 3) no, assumptions of missingness are not discussed. | 1) yes, standardization of different features is explained. 2) no, imputation is not mentioned, therefore it was probably done with complete case analysis. 3) no, imputation was not used. | 1) yes, there is a description of the categorization and meaning of variables. 2) yes, it seems that they have used factorial analysis for mixed data for imputation. 3) no, it is only very shortly mentioned that they used this technique for imputation | 1) no, the pre-processing steps are not mentioned. 2) yes, they used imputation. 3) no, they do not mention details of the imputation or assumptions regarding missingness. |
| **2. Development of the model** | |  |  |  |  |  |  |  |  |
| *2.1 Model selection and interpretability* | 1) Motivation for model selection clearly articulated including potential risks, 2) facilitating interpretability of model, 3) careful selection of number of clusters | 1) yes, they discuss how a random forest works and what the advantages are of this technique. 2) yes, they give an elaborate explanation of what the currently developed model can and cannot do. 3) yes, used the silhouette method for number of cluster selection. | 1) no, rationale for model selection not mentioned. 2) yes, figures show the different characteristics of the clusters. 3) yes, BIC was used. | 1) no, there is no rationale described for selection the k-means clustering algorithm. 2) yes, dendrogram is explained in text and visually supported as well, which supports interpretability of the model. 3) yes, they use a clinical driven approach for number of cluster selection, which means they decided on k=15 clusters because they thought the clusters made most sense this way. | 1) yes, they discuss a rationale for choosing k-means clustering with Gower's distance, which is mostly based on how it handles missing. 2) yes, a figure that illustrates a summary of the study findings is helpful visual aid to understand the model. 3) yes, several statistical measures used to choose ideal cluster amount. | 1) no, motivation for model selection not mentioned. 2) yes, multiple visualizations help understand the outcomes of the model. 3) yes, they used maximum likelihood to decide on number of clusters | 1) yes, they explain the advantages of SOM and claim that this approach makes clustering easier. 2) yes, they provide figures that show the clustering patterns for each comorbidity. 3) yes, applying ward's method. | 1) no, the rationale behind choosing LCA was not reported. 2) yes, easy to understand figures are provided to describe the subgroup characteristics. 3) yes, BIC is used for number of cluster selection | 1) no, they do not mention the rationale for using agglomerative hierarchical clustering. 2) no, they use two different clustering techniques and even with the figures it is hard to understand what the models exactly mean and how they relate to each other. 3) yes, they used elbow method based on distortion score and calinski harabasz score |
| *2.2 Feature selection* | 1) Description of feature selection and feature meaning, 2) removal of highly correlated features, and 3) limiting number of features | 1) yes, they used a supervised learning approach to decide on the 8 most important variables. 2) Yes, using supervised learning to find most important variables will remove highly correlated variables too. 3) Yes, using supervised learning to reduce dimensionality. | 1) no, rationale for feature selection not mentioned. They do explain what every feature means. 2) No, removal of correlated features not described. 3) yes, they used a total of 16 features which is a subset of the original features that are available in this dataset | 1) yes, they describe how the complaint TF-IDF vectors are created and what they mean. 2) no, removal of highly correlated features is not mentioned. 3) no, it is not clear from the paper how many variables were used and whether they reduced this number of variables. | 1) no, there is no description of how they came to their final variable selection. 2) no, checking correlation between variables is not mentioned. 3) no, they mention they included all independent variables and there is no mentioning of limiting the number of variables | 1) no, rationale for variables selection not reported. 2) no, correlation between variables not mentioned. 3) yes, they used a sub selection of all available variables. | 1) yes, they describe that personality traits have a bigger impact on quality of life than LVEF impairment and therefore included these variables. 2) no, correlation is not described. They do have a figure with co-occurring comorbidities which is probably related to correlation, but they do not seem to select features on this information. 3) yes, they ended up selecting six comorbidities for the clustering analysis. | 1) no, they do not explain their feature selection. 2) no, they do calculate correlations between variables but do not select for that. 3) yes, they used a subset of all available variables. | 1) no, it is unclear how many and which variables are included in the analysis. 2) no, it is not mentioned whether they looked at correlation between variables. 3) no, unclear which variables were included. |
| *2.3 Internal validation* | 1) Report measures of internal validation such as reliability or stability of subgroups, 2) meaningfulness of found subgroups, and 3) comparable subgroup sizes with >5% of total number of participants in each subgroup | 1) no, they do not discuss measures that describe the reliability or stability of the subgroups. 2) yes, they discuss which treatment approach would probably be most suitable for each cluster. 3) yes, all groups exceed 5% of total sample size (n = 7358 - 17438) | 1) no, subgroup stability or classification reliability not discussed, and there is no validation step performed. 2) yes, they discuss not only the clinical characteristics of the subgroups but also the differences between the prevalence of each subgroup across Asia. 3) yes, all groups exceed 5% of the total sample size (n = 1048 - 1759) | 1) yes, they use the concept association score to evaluate cluster validity. 2) yes, they dive into the literature to explain cluster characteristics and whether they agree with the literature. 3) no, some clusters have less than 5% of the total sample size (<1297) | 1) yes, they performed a sensitivity analysis by excluding a part of the patients to see whether the same subgroups would be found, 2) yes, they show how found subgroups are a better framework for categorizing heart failure patients. 3) yes, all subgroups exceeded 5% of total sample size (n = 167 - 433) | 1) yes, they performed a sensitivity analysis to evaluate uncertainty in class membership. 2) yes, they discuss whether the patterns of the subgroup characteristics agree with current literature on HF, for example whether it is common that HF patients also suffer from renal failure. 3) no, smallest cluster comprises of 4.7% of total sample size | 1) no, internal validation measures not mentioned. 2) yes, they discuss that their findings are proof for clinical diversity in HF patients, not only physically but also mentally. 3) yes, subgroups all exceeded 5% of total sample size (n = 103 - 183). | 1) yes, they performed sensitivity analysis to check whether the different outcomes of the clusters still existed after correcting for LVEF. 2) yes, they created comorbidity correlation clusters to visualize the interconnectedness of the comorbidities in HF. 3) yes, all subgroups exceed 5% of the total sample size. However, cluster one is very big compared to the others. (n = 666 - 1977) | 1) yes, in the supplement there is a table that shows several statistics regarding stability of the clusters. 2) yes, they do discuss the patterns of the clusters and try to explain them using previous literature. 3) yes, all cluster sizes exceeded 5% of the total sample size (n = 66 - 160) |
| *2.4 Measures to reduce risk of overfitting* | 1) Ratio sample size and candidate features at least 30:1, and 2) penalizing model complexity | 1) yes, after the supervised learning 8 variables are left, and they have more than 8*30 = 240 patients. 2) yes, using silhouette method for number of cluster selection. | 1) yes, more than 16*30 = 480 patients. 2) yes, using BIC for cluster number selection. | 1) no, it is unclear how many variables are used in the final model; therefore, we cannot check this point. 2) no, it seems they only investigated cluster characteristics to decide on number of clusters and did not use a statistic that penalizes model complexity. | 1) yes, they used more than 30*14 = 420 patients. 2) yes, BIC was used. | 1) yes, more than 30*12 = 360 patients, 2) yes, using maximum likelihood to decide on number of clusters | 1) yes, they have more than 6*30 = 180 patients. 2) yes, Ward's method used for cluster selection. | 1) yes, more than 30*12=360 patients. 2) yes, BIC used. | 1) no, unclear how many variables were included in the analysis. 2) yes, using elbow method based on distortion score and calinski harabasz score |
| *2.5 Transparency modelling process* | 1) Reporting software packages and versioning, 2) publishing of the code for the complete model building pipeline, and 3) providing subgroup model algorithm for application | 1) no, packages are mentioned but versioning is missing. 2) no, code for building model pipeline is missing. 3) yes, they provide an online tool where individual patient data can be inserted for cluster assignment and prognostication. | 1) no, versioning is lacking. 2) no, code is not provided. 3) yes, probabilities per comorbidity for subgroup membership is provided. | 1) no, packages and versioning not mentioned. 2) no, code not published. 3) no, no subgroup model algorithm provided. | 1) no, they did not report versioning of the packages. 2) no, code is not provided. 3) no, algorithm is not provided. | 1) no, packages and versioning not mentioned. 2) no, code not provided. 3) yes, probabilities of cluster assignment provided in the supplement. | 1) yes, packages and versioning mentioned. 2) no, code not published. 3) no, subgroup model algorithms not provided. | 1) no, they do mention packaging and versioning however the versioning is not complete. 2) no, code is not published. 3) yes, they provide probabilities of each comorbidity per subgroup. | 1) yes, packages and versioning reported. 2) no, code is not provided. 3) no, algorithm is not provided. |
| **3. Validation of the model** | |  |  |  |  |  |  |  |  |
| *3.1 External validation* | 1) Validation of model in different dataset, and 2) validation performed by independent researchers from other institutions or settings | no external validation | no external validation | no external validation | no external validation | no external validation | no external validation | no external validation | no external validation |
| *3.2 Generalizability* | 1) Validate generalizability of the model using external data from different time period, place, or healthcare setting, 2) validate in dataset with sufficient sample size | no external validation | no external validation | no external validation | no external validation | no external validation | no external validation | no external validation | no external validation |
